# Enantioselective transformation of phytoplankton-derived dihydroxypropanesulfonate by marine bacteria

**DOI:** 10.1093/ismejo/wrae084

**Published:** 2024-05-06

**Authors:** Le Liu, Xiang Gao, Changjie Dong, Huanyu Wang, Xiaofeng Chen, Xiaoyi Ma, Shujing Liu, Quanrui Chen, Dan Lin, Nianzhi Jiao, Kai Tang

**Affiliations:** State Key Laboratory of Marine Environmental Science, Fujian Key Laboratory of Marine Carbon Sequestration, College of Ocean and Earth Sciences, Xiamen University, Xiang'an South Road, Xiamen 361102, China; State Key Laboratory of Cellular Stress Biology, Fujian Provincial Key Laboratory of Innovative Drug Target Research, School of Pharmaceutical Sciences, Xiamen University, Xiamen 361102, China; State Key Laboratory of Marine Environmental Science, Fujian Key Laboratory of Marine Carbon Sequestration, College of Ocean and Earth Sciences, Xiamen University, Xiang'an South Road, Xiamen 361102, China; State Key Laboratory of Marine Environmental Science, Fujian Key Laboratory of Marine Carbon Sequestration, College of Ocean and Earth Sciences, Xiamen University, Xiang'an South Road, Xiamen 361102, China; Technical Innovation Center for Utilization of Marine Biological Resources, Third Institute of Oceanography, Ministry of Natural Resources, Xiamen 361001, China; State Key Laboratory of Marine Environmental Science, Fujian Key Laboratory of Marine Carbon Sequestration, College of Ocean and Earth Sciences, Xiamen University, Xiang'an South Road, Xiamen 361102, China; State Key Laboratory of Marine Environmental Science, Fujian Key Laboratory of Marine Carbon Sequestration, College of Ocean and Earth Sciences, Xiamen University, Xiang'an South Road, Xiamen 361102, China; State Key Laboratory of Marine Environmental Science, Fujian Key Laboratory of Marine Carbon Sequestration, College of Ocean and Earth Sciences, Xiamen University, Xiang'an South Road, Xiamen 361102, China; State Key Laboratory of Marine Environmental Science, Fujian Key Laboratory of Marine Carbon Sequestration, College of Ocean and Earth Sciences, Xiamen University, Xiang'an South Road, Xiamen 361102, China; State Key Laboratory of Marine Environmental Science, Fujian Key Laboratory of Marine Carbon Sequestration, College of Ocean and Earth Sciences, Xiamen University, Xiang'an South Road, Xiamen 361102, China; State Key Laboratory of Marine Environmental Science, Fujian Key Laboratory of Marine Carbon Sequestration, College of Ocean and Earth Sciences, Xiamen University, Xiang'an South Road, Xiamen 361102, China

**Keywords:** organosulfur cycle, bacteria, ocean, *Roseobacteraceae*, phytoplankton, chirality

## Abstract

Chirality, a fundamental property of matter, is often overlooked in the studies of marine organic matter cycles. Dihydroxypropanesulfonate (DHPS), a globally abundant organosulfur compound, serves as an ecologically important currency for nutrient and energy transfer from phytoplankton to bacteria in the ocean. However, the chirality of DHPS in nature and its transformation remain unclear. Here, we developed a novel approach using chiral phosphorus-reagent labeling to separate DHPS enantiomers. Our findings demonstrated that at least one enantiomer of DHPS is present in marine diatoms and coccolithophores, and that both enantiomers are widespread in marine environments. A novel chiral-selective DHPS catabolic pathway was identified in marine *Roseobacteraceae* strains, where HpsO and HpsP dehydrogenases at the gateway to DHPS catabolism act specifically on *R*-DHPS and *S*-DHPS, respectively. *R*-DHPS is also a substrate for the dehydrogenase HpsN. All three dehydrogenases generate stable hydrogen bonds between the chirality-center hydroxyls of DHPS and highly conserved residues, and HpsP also form coordinate–covalent bonds between the chirality-center hydroxyls and Zn^2+^, which determines the mechanistic basis of strict stereoselectivity. We further illustrated the role of enzymatic promiscuity in the evolution of DHPS metabolism in *Roseobacteraceae* and SAR11. This study provides the first evidence of chirality’s involvement in phytoplankton-bacteria metabolic currencies, opening a new avenue for understanding the ocean organosulfur cycle.

## Introduction

The major building blocks of life, such as amino acids and sugars, are typically optically pure, meaning they are composed of a single enantiomer, creating a homochiral world [1, 2]. However, chiral molecules in living organisms, such as some secondary metabolites [3, 4], may exist with their mirror images as heterochiral mixtures. Enantiomeric secondary metabolites may not interfere with the primary metabolic functions essential for microbial cell growth and development [3], but different configurations of chiral enantiomers may significantly influence the biological activities towards microorganisms, likely improving microbial survival [5]. The ocean is expected to be a rich source of naturally occurring chiral molecules due to the potential of diverse marine organisms to produce a vast array of metabolites [6]. However, current knowledge of the natural pairs of enantiomers in the marine environment is limited to amino acids. d-amino acids are primarily derived from bacterial cell walls and far less abundant than l-amino acids [7–9]. The reason of enantioselectivity is that living organisms are formed of homochiral structural units of l-amino acids, leading to the persistence of d-isomers in the ocean for a long time as a potential component of recalcitrant dissolved organic carbon [8].

Phytoplankton are the primary producers of organic matter in the ocean, accounting for half of all photosynthesis on Earth [10]. Their metabolites, such as sugars, amino acids, and sulfur-containing compounds, serve as metabolic currencies in the surface ocean to support the microbial loop, substantially fueling the nutrient needs of heterotrophic bacteria [11–13]. Most research on marine organic matter cycling has focused on phytoplankton–bacteria interactions, metabolic pathways, and organic matter composition [13–16], neglecting the chiral resolution and absolute configuration determination of organic compounds. Chiral dihydroxypropanesulfonate (DHPS), one of the most abundant organosulfur in the biosphere, can be derived from microbial degradation of sulfoquinovose [17], a ubiquitous compound in photosynthetic organisms with an estimated annual global production of 10 billion tonnes [17]. Moreover, DHPS can reach millimolar intracellular concentrations in some phytoplankton cells, comparable to the widespread dimethylsulfoniopropionate (DMSP), and serve as an ecologically important metabolic currency for heterotrophic bacteria in the ocean [18, 19]. DHPS plays a vital role in linking the carbon and sulfur cycles [19]. Phytoplankton may secrete DHPS to marine bacteria, such as prevalent *Roseobacteraceae*, as a source of carbon and sulfur, and in return, these bacteria provide the essential vitamin B_12_ to the phytoplankton [19]. Our previous study revealed that marine *Roseobacteraceae* strains can completely catabolize both enantiomers of DHPS in batch cultures using a DHPS-degrading gene cluster [20]. However, the enantiomeric composition of DHPS in nature has remained elusive and the understanding for chiral DHPS catabolic pathway is hindered by the enantioselectivity of enzymes. In this study, we developed a chiral phosphorus reagent labelling approach to determine the enantiomers of DHPS in phytoplankton cells and marine environments. We identified enantioselective enzyme reactions and further constructed the chiral DHPS catabolic pathway in *Roseobacteraceae* strains through biochemical analysis and high-resolution crystal structures. Additionally, we evaluated the enzymatic promiscuity of DHPS dehydrogenases and their homologs by examining their catalytic activities towards a range of substrates. This study sheds light on a previously overlooked aspect of the marine organosulfur cycle, revealing the significance of chirality in phytoplankton-bacteria metabolic currencies.

## Materials and methods

### Culture conditions of phytoplankton and bacterial strains

Phytoplankton strains were obtained from Center for Collections of Marine Algae (Xiamen University, China). Diatoms and haptophytes were grown under the following conditions: light of 100 μE·m^−2^·s^−1^ and temperature of 20°C. Diatoms were incubated in f/2 media [21] supplemented with Na_2_SiO_3_. Haptophytes were incubated in f/2 media without Na_2_SiO_3_. Cell abundance was measured using an Accuri C6 flow cytometer (BD Biosciences) or microscopy. Samples of phytoplankton cultures at stationary phase were collected. Cell volumes of *Skeletonema costatum* 125, *Thalassiosira weissflogii* 102, *Chaetoceros gracilis* 116, *Chaetoceros debilis* Cleve 117, *Ditylum brightwelli* 108, and *Gephyrocapsa oceanica* were based on cell dimensions directly observed under the microscope and calculated using a previously reported method [22]. Cell volumes of *Navicula pelliculosa* 350, *Thalassiosira pseudonana* 220, *Phaeodactylum tricornutum* 267, *Emiliania huxleyi* 1516, *E. huxleyi* 2090, and *E. huxleyi* CS369 were taken from previous descriptions [23]. The bacterial strains *Dinoroseobacter shibae* DFL 12 (DSM 16493) and *Ruegeria pomeroyi* DSS-3 (DSM 15171) were obtained from German Collection of Microorganisms and Cell Cultures (Braunschweig, Germany). *D. shibae* DFL 12 were grown at 28°C in modified Silicibacter basal medium [24] (pH 7.2) containing 10 mM DHPS or 15 mM acetate. *R. pomeroyi* DSS-3 were grown at 28°C in modified Silicibacter basal medium (pH 7.2) containing 10 mM DHPS or 15 mM acetate supplemented with 0.25 g/L yeast extract. The samples of bacteria cultures were collected at stationary phase.

### Study sites and field sampling

Samples were collected from 19 sites in the Bohai Sea, Yellow Sea, East China Sea, and South China Sea. Seawater was collected from a depth of 3 m below the surface using Niskin bottles. Seawater (1–2 L) was pre-filtered through a nylon screen (200 μm), then filtered via a precombusted (480°C for 5 h) GF/F glass fiber filter membrane (47 mm, 0.75 μm, Whatman). The filters were subsequently stored at −80°C until further analysis.

### Synthesis of *R*-, *S*-DHPS and diisopropyl phosphoryl l-alanine

*R*-, *S*-DHPS were synthesized chemically and verified according to previous protocols [20]. The chiral phosphorus reagent diisopropyl phosphoryl l-alanine (P-l-Ala) was synthesized using the recently published protocol [25]. The structure of P-l-Ala was verified by ^1^H NMR (Supplementary Fig. S1). P-l-Ala: ^1^H NMR: (400 MHz, Chloroform-d) *δ* 9.41 (s, 1H), 4.78 (t, *J* = 9.4 Hz, 1H), 4.61 (dddd, *J* = 20.0, 13.7, 12.3, 6.1 Hz, 2H), 3.86 (h, *J* = 7.1 Hz, 1H), 1.46 (d, *J* = 7.1 Hz, 3H), 1.39–1.18 (m, 12H) ppm.

### The labelling assay of *R*-*, S*-DHPS

A pair of enantiomers share identical physicochemical properties in an achiral environment [3], making them cannot be separated on achiral columns. The most common strategy for separating enantiomers is using chiral high performance liquid chromatography (HPLC) [3]. However, chiral HPLC method could not be applied to the separation of *R*-DHPS and *S*-DHPS. Additionally, organic matter compounds exist generally very low concentrations in the ocean and have complex molecular environments [19], making it a challenge for characterizing the chiral organic molecules in field samples. The phosphorus reagents such as chiral P-L-Ala and H-phosphite can significantly improve the detection sensitivity of metabolites in mass spectrometry [25, 26]. We used the phosphorus reagent chiral P-l-Ala to label the secondary alcohol of *R*-, *S*-DHPS to convert enantiomers into diastereomers, which were separated on regular C18 column. To label *R*-, *S*-DHPS, 15 mg of DHPS (*R*-DHPS, *S*-DHPS or racemic DHPS, 0.1 mmol) was mixed with 49 mg P-l-Ala (0.2 mmol), 83 mg of dicyclohexylcarbodiimide (DCC, 0.4 mmol), and 49 mg of 4-(Dimethylamino) pyridine (DMAP, 0.4 mmol) in 1 ml dichloromethane in a 5 ml round-bottom flask. The flask was sealed with a rubber stopper and the mixture was reacted at room temperature for 12 h with magnetic stirring. Next, the dichloromethane was removed by rotary evaporation, and the precipitate was redissolved in 1 ml of methanol. The solution was vortexed and centrifuged at 12 000 rpm for 5 min to remove any precipitate. The supernatant was then analyzed by HPLC-MS/MS (Supplementary Fig. S2). To label *R*-, *S*-DHPS extracted from cultured phytoplankton and field samples, the metabolites were first extracted according to the previously published protocol [23]. The extracted metabolites were dried using rotary evaporation. The dry weight of extracted metabolites was used as the weight of total *R*-, *S*-DHPS to calculate the amounts of P-l-Ala, DCC, and DMAP (*R*-, *S*-DHPS: P-l-Ala: DCC: DMAP = 1: 2: 4: 4, n: n: n: n), required to make sufficient reagent for the labelling of *R*-, *S*-DHPS. The dry metabolites were redissolved in 1 ml of water and the precipitates were removed by centrifugation at 12 000 rpm for 5 min. The supernatant was then adjusted to pH 2.0 with formic acid and loaded onto a Hypersep NH_2_ column (500 mg/3 ml, Thermo Fisher Scientific). The column was washed with 3 ml of methanol to remove any nonpolar metabolites. *R*-, *S*-DHPS were eluted with 3 ml of triethylammonium bicarbonate buffer (1 M, pH 8.5, Sigma-Aldrich). The extracted *R*-, *S*-DHPS solutions were dried using rotary evaporation and then redissolved in 1 ml dichloromethane. P-l-Ala, DCC, and DMAP were proportionally added to the reaction mixture with extracted *R*-, *S*-DHPS in a 5 ml round-bottom flask. The subsequent steps were the same as the labeling procedures for the standards of *R*-, *S*-DHPS described above. The quantification of total *R*-, *S*-DHPS was performed according to the previously published protocol [23].

### Identification of labeled *R*-, *S*-DHPS by HPLC-MS/MS

The identifications of labeled *R*-, *S*-DHPS were performed using a Q Exactive high performance liquid chromatography system (Thermo Fisher Scientific, Waltham, USA) equipped with a Hypersil Gold C18 column (5 μm, 200 Å, 150 × 2.1 mm; Thermo Fisher Scientific) coupled to an orbitrap mass spectrometer (Thermo Fisher Scientific). Electrospray ionization was performed in negative mode ionization with the following parameters: capillary temperature 320°C, spray voltage 3500 V, sheath gas flow 40 arbitrary units and aux gas flow 10 arbitrary units. The HPLC conditions were as follows: the column was equilibrated with 95% solvent A (10 mM NH_4_Ac) and 5% solvent B (100% acetonitrile) for 5 min. The gradient was comprised of an increase from 5% to 50% solvent B over 40 min, and increase to 90% solvent B over 10 min, and held at 90% solvent B for 5 min. Then, it was decreased from 90% to 5% solvent B over 5 min, and finally held at 5% solvent B for 7 min. The flow rate was 0.25 ml/min and the injection volume was 2 μl.

### Heterologous expression and purification of DHPS catabolic enzymes

Genes were heterologously expressed in *Escherichia coli* BL21(DE3) harboring the plasmid pET28a. The mutants were created using the site-directed mutagenesis kit from Tiangen Biotech. The primer pairs used for site-directed mutagenesis are presented in Supplementary Table S1. *E. coli* BL21(DE3) was grown in lysogeny broth at 37°C with 200 rpm shaking until the optical density (OD_600_) of the cultures reached 0.8. The cultures were then cooled to 16°C and isopropyl β-d-1-thiogalactopyranoside was added to a finial concentration of 0.5 mM. The cells were further incubated at 16°C with 200 rpm shaking for 20 h. The cultures were centrifuged at 12 000 g for 10 min at 4°C. The cell pellets were resuspended in 50 mM Tris–HCl buffer (pH 8.0, 150 mM NaCl). The suspension was subjected to ultrasonication to disrupt the cells. The cell lysate was then centrifuged at 12 000 g for 20 min at 4°C. The supernatants were filtered through a 0.45-μm membrane (Merck Millipore) and then loaded onto AKTA pure (Cytiva, MA, USA) coupled with a HisTrap HP column (5 ml, Cytiva). The product fraction was eluted with imidazole-containing (50–500 mM) elution buffer (10 mM Tris–HCl, 150 mM NaCl, pH 8.0) at a flow rate of 1 ml/min. The collected product fractions were further subjected to a desalting column (5 ml, Cytiva) with elution buffer to remove imidazole.

### Determination of the metals in proteins

To investigate the metals present in HpsP from *D. shibae* DFL 12 (*Ds*HpsP, locus tag: Dshi_2936) and HpsN from *R. pomeroyi* DSS-3 (*Rp*HpsN, SPO0594), we performed inductively coupled plasma-mass spectrometry (ICP-MS) analysis. *Ds*HpsP and *Rp*HpsN were subjected to the purification steps described above before metals analysis in ICP-MS. Homogeneous enzymes were digested according to the modified protocol [27]. A total of 400 μl of nitric acid (inorganic trace analysis grade, Suprapur, Germany) were added to 400 μl of protein samples and samples were incubated at 70°C for 5 h. Digested protein samples were diluted with ddH_2_O to 20 ml. The metal contents were analyzed by ICP-MS (NexION 2000, PerkinElmer).

### Determination of the affinities of DHPS dehydrogenases towards *R*-, *S*-DHPS using surface plasmon resonance (SPR)

SPR-based assay was used to determine the affinities of *R*-, *S*-DHPS towards DHPS dehydrogenases on Biacore T200 (Cytiva). The purified proteins *Rp*HpsO (SPO0595), *Rp*HpsP (SPO0596), and *Rp*HpsN were first subjected to a gel-filtration column (Superdex 200 Increase 10/300 GL, Cytiva) with phosphate buffer (10 mM, pH 8.0) and then concentrated to ~1 mg/ml using ultrafiltration tubes (3000 Dalton, 1.5 ml, Merk). The proteins were immobilized on CM5 sensor chips (Cytiva) by standard amine-coupling method at 25°C with running buffer (phosphate, 10 mM, pH 8.0). The carboxyl groups on the sensor surface were activated by injection of a solution containing 0.2 M N-ethyl-N9-(3-dimethylaminopropyl) carbodiimide and 0.05 M N-hydroxysuccinimide. *Rp*HpsO, *Rp*HpsP, and *Rp*HpsN in sodium acetate buffer (pH 3.5) were then injected to couple to the senor surfaces with a flow rate of 30 μl/min. The remaining carboxyl groups on the sensor surface were finally blocked with 1 M ethanolamine. The reference flow cell was activated and blocked in the absence of proteins. *R*-DHPS was injected into the flow cell fixed with *Rp*HpsO at concentrations of 0.0625, 0.125, 0.2, 0.25, 0.312, 0.5, and 1.0 mM. *S*-DHPS was injected into the flow cell fixed with *Rp*HpsP at concentrations of 0.078, 0.156, 0.312, 0.625, 1.25, and 2.5 mM. *R*-DHPS was injected into the flow cell fixed with *Rp*HpsN at concentrations of 0.078, 0.156, 0.312, 0.625, 1.25, 2.5, and 5.0 mM. The equilibrium dissociation constants (*K*_D_) of *R*-DHPS and *S*-DHPS were obtained by fitting the data sets to 1:1 Langmuir binding model using Biacore T200 Evaluation Software.

### Enzyme assay

Enzymatic reaction mixture (250 μl) contained 10 mM Tris–HCl pH 8.0, 150 mM NaCl, 10 mM NAD^+^ or NADP^+^, 10 mM DHPS or sulfolctaldehyde, and enzymes. Metal chelating agents 1, 10-phenanthroline (10 mM) and ethylene diamine tetraacetic acid (EDTA, 1 mM) were incubated with *Ds*HpsP and *Rp*HpsN for 30 min, respectively, followed by dialysis using 10 000-Dalton membranes (Solarbio, Beijing, China) to remove the chelated metals. To investigate the metals dependence of *Ds*HpsP and *Rp*HpsN, the assay buffer containing 50 μM ZnCl_2_ was applied. To make sure every mutant sample of *Ds*HpsP and *Rp*HpsN containing excess metal ions in reaction mixtures, the activities of mutants were assayed in buffer containing 50 μM ZnCl_2_. Enzymatic activities were assayed spectrophotometrically as the formation of the coproduct NADH/NADPH [28]. The reaction mixtures were incubated in 96-well plates at 30°C for 4 min, and the absorbance at 340 nm was monitored using a SynergyH1 Multi-Mode Reader (BioTek, Vermont, USA).

### High performance liquid chromatography-MS/MS analysis

The enzymatic reaction products were analyzed by HPLC-MS/MS. The reaction systems were the same as above-described mixtures, except that the buffer contained 200 mM ammonium formate pH 8.0 without NaCl. This is because Tris and NaCl can suppress the signal response on mass spectrometer detectors in electrospray ionization process during mass spectrometry. The reaction mixtures were ultra-filtered to remove enzymes using Amicon Ultra-0.5 device (3000 Dalton, 0.5 ml, Merck). A total of 100-μl of filtrate was taken and added to 900-μl acetonitrile. The mixture was then filtered through a 0.22-μm nylon membrane into a 1-ml glass vial. The reaction products were analyzed on a Q Exactive HPLC system (Thermo Fisher Scientific) fitted with a ZIC-HILIC column (3.5 μm, 200 Å, 150 × 2.1 mm; Merck). The column was connected to an orbitrap mass spectrometer (Thermo Fisher Scientific). Electrospray ionization was performed in negative mode ionization with the following parameters: capillary temperature 320°C, spray voltage 3500 V, sheath gas flow 40 arbitrary units and aux gas flow 10 arbitrary units. The HPLC conditions were as follows: the column was equilibrated with 90% solvent A (100% acetonitrile) and 10% solvent B (10 mM NH_4_Ac) for 10 min. The gradient was comprised of an increase from 10% to 35% solvent B over 25 min, then held at 35% solvent B for 10 min, 35% to 10% solvent B over 0.5 min, and finally held at 10% solvent B for 10 min. The flow rate was 0.25 ml/min and the injection volume was 2 μl. The detections of DHPS, sulfolactaldehyde, and sulfolactate in phytoplankton and bacteria use the same parameters of electrospray ionization and HPLC conditions described above. For the detection of cysteinolic acid and 2-oxo-3-hydroxy-propane-1-sulfonate in phytoplankton, an UHPLC BEH Amide column (1.7 μm, 2.1 mm × 100 mm, Waters, MA, USA) was used. The HPLC conditions were as follows: the column was equilibrated with 90% solvent A (100% acetonitrile) and 10% solvent B (10 mM NH_4_Ac) for 10 min. The gradient was comprised of an increase from 10% to 35% solvent B over 15 min, then further increased to 60% solvent B over 1 min, held at 60% solvent for 4 min, 60% to 10% solvent B over 1 min and finally held at 10% solvent B for 10 min. The flow rate was 0.25 ml/min and the injection volume was 2 μl. Electrospray ionization was performed in negative and positive mode ionization as described above.

### Protein crystallization

The proteins purified with nickel were further subjected to gel filtration column (Superdex 200 Increase 10/300 GL, Cyvita), and the protein solutions were concentrated to 10 mg/ml. The initial screens of the crystals were performed in 96-well plates using the sitting-drop vapor diffusion method. Commercial crystal screen kits from Hampton Research, Qiagen, Molecular Dimensions, and Greiner were used. The hanging-drop method was used for crystal optimization in a 24-well plate. The crystal of *Rp*HpsO was obtained at 20°C with the condition of 0.1 M sodium citrate pH 5.4 and 0.8 M sodium formate. Because the protein crystal of *Rp*HpsP could not be obtained, we turned to *Ds*HpsP, which was crystallized at 18°C with the condition of 0.2 M sodium formate and 18% PEG 3350. The crystal of *Rp*HpsN was obtained at 18°C with the condition of 0.1 M Tris pH 7.5 and 1.1 M ammonium sulfate. Crystals were harvested with nylon CryoLoops (Hampton Research) and cryopreserved in liquid nitrogen with 20% glycerol as the cryoprotectant.

### X-ray data collection, processing, and refinement

Diffraction data were collected from a single crystal of each protein at the Shanghai Synchrotron Radiation Facility BL18U beamline, China, with a wavelength of 0.97915 Å at 100 K. The diffraction data were processed and scaled with HKL-3000 [29]. The resolution of *Rp*HpsO structure was solved at 1.7 Å utilizing a molecular replacement method using protein data bank (PDB) entry 3O03 [30] as an initial search model. The resolution of *Ds*HpsP structure was solved at 2.3 Å utilizing a molecular replacement method using PDB entry 1E3J [31] as an initial search model. The crystal of *Rp*HpsN could be obtained, but the atom coordinates of the structure could not be solved by molecular replacement using the protein structures in PDB database. Therefore, we predicted the structure of *Rp*HpsN by AlphaFold2 [32] and removed domain A (Val218 to Leu374) of the predicted *Rp*HpsN structure. The truncated structure was used as template to successfully solve the crystal structure of *Rp*HpsN with 2.9 Å. Structures were built and refined by iterative cycles using Coot [33] and Phenix [34]. Stereochemical qualities of the structures were checked using PROCHECK. The statistics for data collection and final refinement are presented in Supplementary Table S2. The dimer structure of *Rp*HpsN was shown in Supplementary Fig. S3A. AlphaFold2-predicted structure of *Rp*HpsN was very similar to l-histidinol dehydrogenase from *E. coli* MC1061 (PDB ID: 1KAE), with RMSD value of 1.45 Å (Supplementary Fig. S3B). However, domain A in the crystal structure of the *Rp*HpsN showed significant difference in spatial position compared to the AlphaFold2-predicted structure of *Rp*HpsN as well as structure of l-histidinol dehydrogenase (Supplementary Fig. S3B). RMSD value was 1.27 Å between domain A of the *Rp*HpsN crystal structure and domain A of l-histidinol dehydrogenase (Supplementary Fig. S3C). The lack of electron density for the amino acid segments Val218-Gly225 and Thr348-Leu374 at the junction between domain A and domain B in *Rp*HpsN crystal structure suggests a high flexibility in this region. Thus, the position of domain A in the *Rp*HpsN structure was further optimized using the AlphaFold2-predicted structure, and the optimized *Rp*HpsN structure was used for subsequent analysis (Supplementary Fig. S3D).

### Molecular docking and dynamics simulation

For *Rp*HpsO structure, the structure of 2-keto-3-deoxy-d-gluconate dehydrogenase complexed with NAD^+^ (PDB ID: 4ZA2) [35] was used as a template to superimpose with the *Rp*HpsO structure. The RMSD between the structures was 1.32 Å over 238 residues. The structure of 2-keto-3-deoxy-d-gluconate dehydrogenase was then removed, and the structure of *Rp*HpsO with NAD^+^ was retained for molecular docking by *R*-DHPS. For *Ds*HpsP structure, threonine 3-dehydrogenase complexed with NAD^+^ (PDB ID: 2EJV) was used as template to superimpose with the *Ds*HpsP structure. The RMSD between the structures was 1.8 Å over 231 residues. The structure of threonine 3-dehydrogenase was then removed, and the structure of *Ds*HpsP with NAD^+^ were retained for molecular docking by *S*-DHPS. For *Rp*HpsN structure, l-histidinol dehydrogenase complexed with NAD^+^ (PDB ID: 1KAE) [36] was used as template to superimpose with the optimized *Rp*HpsN structure. The value of RMSD between the structures was 1.6 Å over 377 residues. The structure of l-histidinol dehydrogenase was then removed, and the structure of *Rp*HpsN with NAD^+^ and Zn^2+^ were retained for molecular docking by *R*-DHPS. The tool US align [37] was used to superimpose the protein structures. Molecular docking was performed using the tool AutoDock 4 tool [38]. Proteins and ligands were added by gasteiger charges and all hydrogen atoms. Genetic algorithm was used, and the maximum number of evals was set to medium. The generated conformations with the lowest binding energy were retained for further molecular dynamics simulation analysis.

The molecular dynamics simulations for the docked models of *Rp*HpsO, *Ds*HpsP, and *Rp*HpsN were performed using the NAMD program [39]. For *Rp*HpsO, the complex model was placed in an orthorhombic box with 15 711 water molecules, 55 sodium ions, and 42 chloride ions. For *Ds*HpsP, the complex model was placed in an orthorhombic box with 7878 water molecules, 25 sodium ions, and 21 chloride ions. For *Rp*HpsN, the complex model was placed in an orthorhombic box with 15 730 water molecules, 55 sodium ions, and 42 chloride ions. Sodium and chloride ions were randomly spread under periodic boundary conditions. The solvation systems were minimized during the 1 ns, and the equilibrated systems were followed by 30 ns molecular dynamics at 300 K. The protein structures were visualized with Pymol (http://www.pymol.org/).

### Bioinformatic analysis

For phylogenetic analysis, the sequences of *hpsO, hpsP*, and *hpsN* were searched against NCBI Reference Sequence Database using the clinker tool [40]. The genomes that simultaneously contained all three genes were retained. A phylogenetic tree for the distribution of candidate *hpsO*, *hpsP*, and *hpsN* was constructed using the concatenated alignment of nine single-copy orthologous genes (*rnc*, *rplC*, *rplF*, r*plS*, *rplV*, *rpoA*, *rpsH*, *rpsJ*, and *smpB*) shared by all bacterial strains. The tree was constructed using the Maximum Likelihood method using the RAxML program [41] and the LG + IU + G4m model. Bootstrap resampling was performed for 1000 replications. Phylogenetic trees were visualized with the Evolview v3 program [42]. For gene transcripts analysis in *Tara* Oceans, the sequences of *R*-, *S*-DHPS dehydrogenases from *R. pomeroyi* DSS-3 were blasted against OM-RGC_v2_metaT datasets in the *Tara* Oceans database [43]. The bit scores of 167 for HpsN, 145 for HpsO, and 137 for HpsP were used as cut-off values, which were determined according to previously published protocol [23]. The gene transcripts abundance was the homologs fraction in the total gene set for each sample, which were presented as reads per kilobase million. The abundance of eukaryotes and cyanobacteria were obtained from *Tara* Oceans OTU 18S V9 version 2 database [44]. Correlation analysis was performed by Origin 2021 (OriginLab, MA, USA). The protein structural homologues search used Foldseek [45]. All of the predicted protein structures were predicted by AlphaFold2 [32].

## Results

### Chiral distribution of DHPS in marine phytoplankton and environments

**Figure 1:**
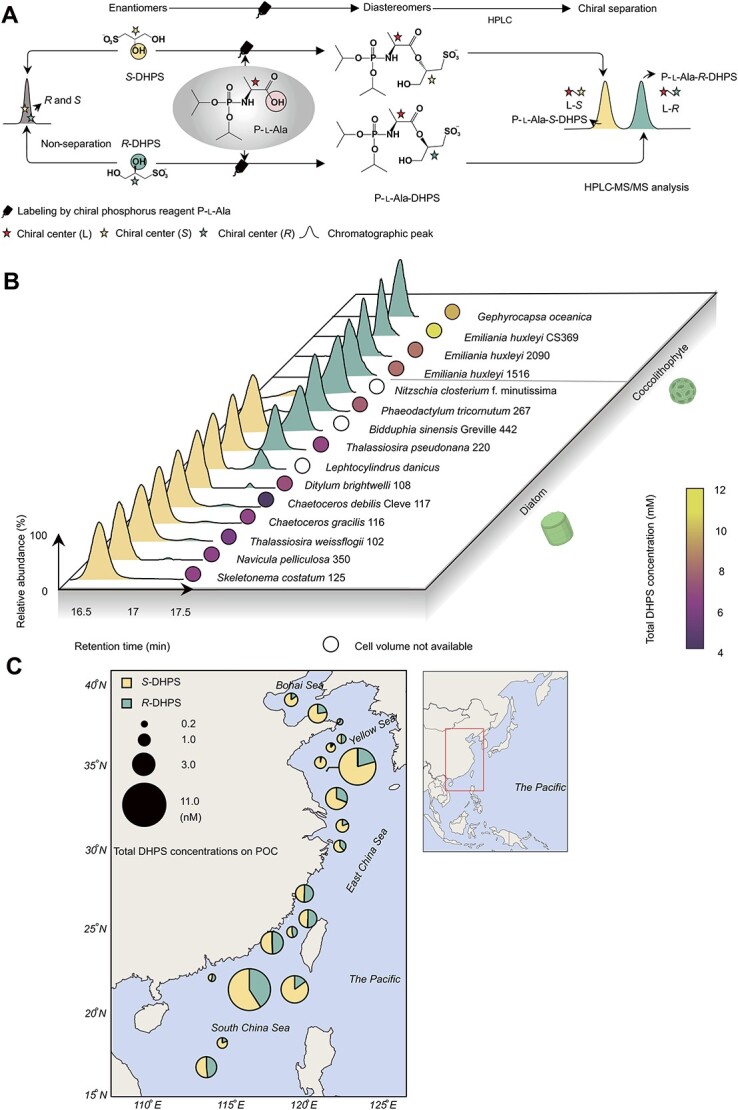
Chiral separation and distribution of *R*-DHPS and *S*-DHPS in phytoplankton and seawater. (**A**) Schematic of the labelling of *R-* and *S*-DHPS by P-l-Ala and the separation via HPLC. Esterification between the C2-OH of *R*-, *S*-DHPS and carboxyl group of P-l-Ala converts the enantiomers to labeled diastereomers. The chromatographic peak of labeled *S*-DHPS (P-l-Ala-*S*-DHPS) has a relative shorter retention time compared to that of labeled *R*-DHPS (P-l-Ala-*R*-DHPS) on C18 column. (**B**) The distributions of *R*-DHPS and *S*-DHPS in diatoms and haptophytes. The chromatographic peaks represent the P-l-Ala-labeled *R-* and *S*-DHPS extracted from phytoplankton cells. The color of filled circles represent the concentration levels of total *R-* and *S*-DHPS within these phytoplankton cells. Empty circles indicate that the total *R-* and *S*-DHPS concentrations within these phytoplankton cells could not be calculated as cell volume was unknown. (**C**) The distributions of *R*-DHPS and *S*-DHPS in Bohai Sea, Yellow Sea, East China Sea, and South China Sea. The sizes of circles represent the total *R-* and *S*-DHPS concentrations in particulate organic carbon (POC). The mole ratios of *R*-DHPS to *S*-DHPS in the samples are represented as sectors in the circles.

We developed a novel separation strategy for DHPS enantiomers, by labeling the chiral center C2-OH of *R*-, *S*-DHPS with the chiral phosphorus reagent P-l-Ala. This converted *R*-, *S*-DHPS enantiomers into non-enantiomeric molecules (Fig. 1A). The introduction of a neutral phosphate group increases the overall hydrophobicity of targeted metabolites and ionization efficiency in electrospray ionization processes, leading to better chromatographic separation of hydrophilic metabolites on regular reversed-phase columns and enhanced signal response on mass spectrometer detectors [25]. Labeled *R*-DHPS and *S*-DHPS achieved baseline separation on a C-18 column (Fig. 1A). The most common diatom and coccolithophore species in the coastal waters of China can produce enantiomers of DHPS with intracellular concentrations reaching millimolar level (4–12 mM), making DHPS one of the most abundant organosulfur metabolites in phytoplankton (Fig. 1B and Supplementary Data 1). Only *R*-DHPS was detected in all four investigated coccolithophores strains (Fig. 1B and Supplementary Data 1). In diatoms, both *R*-DHPS and *S*-DHPS were detected as intracellular metabolites (Fig. 1B and Supplementary Data 1). *S*-DHPS was notably higher than *R*-DHPS in most (7/11) investigated diatom strains, except for *Bidduphia sinensis* Greville and *T. pseudonana*, which had roughly equal amounts of the two enantiomers, and in *Nitzschia closterium* f. minutissima and *P. tricornutum*, which produced more *R*-DHPS than *S*-DHPS. Both DHPS enantiomers were also widely detected in surface seawater particulates from coastal areas along China, with total DHPS concentrations ranging from 0.2 to 11 nM (Fig. 1C and Supplementary Data 1). The two DHPS enantiomers were approximately equivalent in most samples from the East and South China Seas (8/10 samples), while *S*-DHPS was predominant in most samples from the North China Sea (7/9 samples). The variation in the enantiomeric ratios of DHPS in marine environments may be associated with differences in phytoplankton species composition, especially that of diatoms, which are often the dominant group in the coastal waters of China [46–49].

### Reconstructed pathway for DHPS catabolism in bacteria

**Figure 2:**
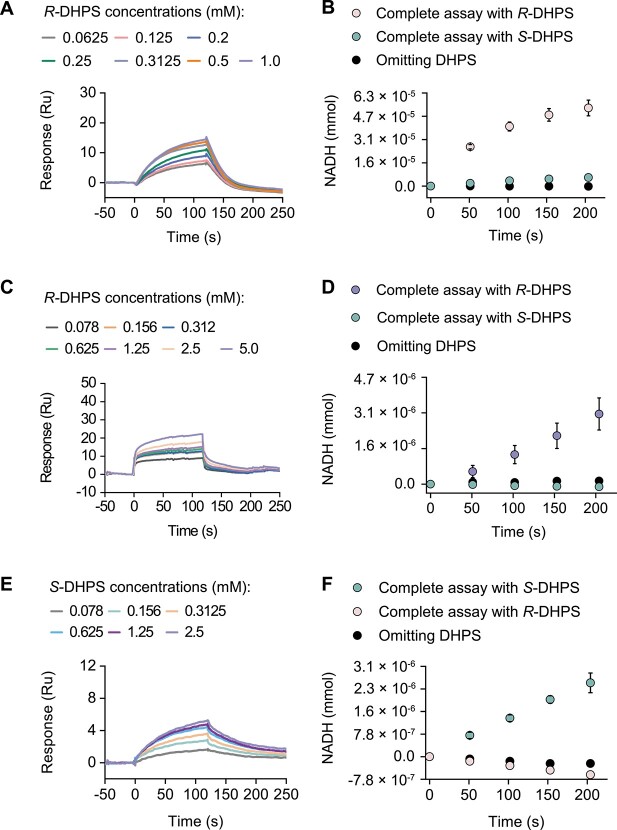
The binding and oxidation of *R-* and *S*-DHPS by DHPS dehydrogenases. (**A**) SPR sensorgrams of *Rp*HpsO binding *R*-DHPS with *K*_D_ value of 0.33 mM. (**B**) Enzymatic activity assay monitoring NADH formation accompanying *R*-DHPS oxidation by *Rp*HpsO (0.4 μM). (**C**) SPR sensorgrams of *Rp*HpsN binding *R*-DHPS with *K*_D_ value of 0.86 mM. (**D**) Enzymatic activity assay monitoring NADH formation accompanying *R*-DHPS oxidation by *Rp*HpsN (0.4 μM). (**E**) SPR sensorgrams of *Rp*HpsP binding *S*-DHPS with *K*_D_ value of 0.74 mM. (**F**) Enzymatic activity assay monitoring NADH formation accompanying *S*-DHPS oxidation by *Rp*HpsP (0.5 μM).

**Figure 3:**
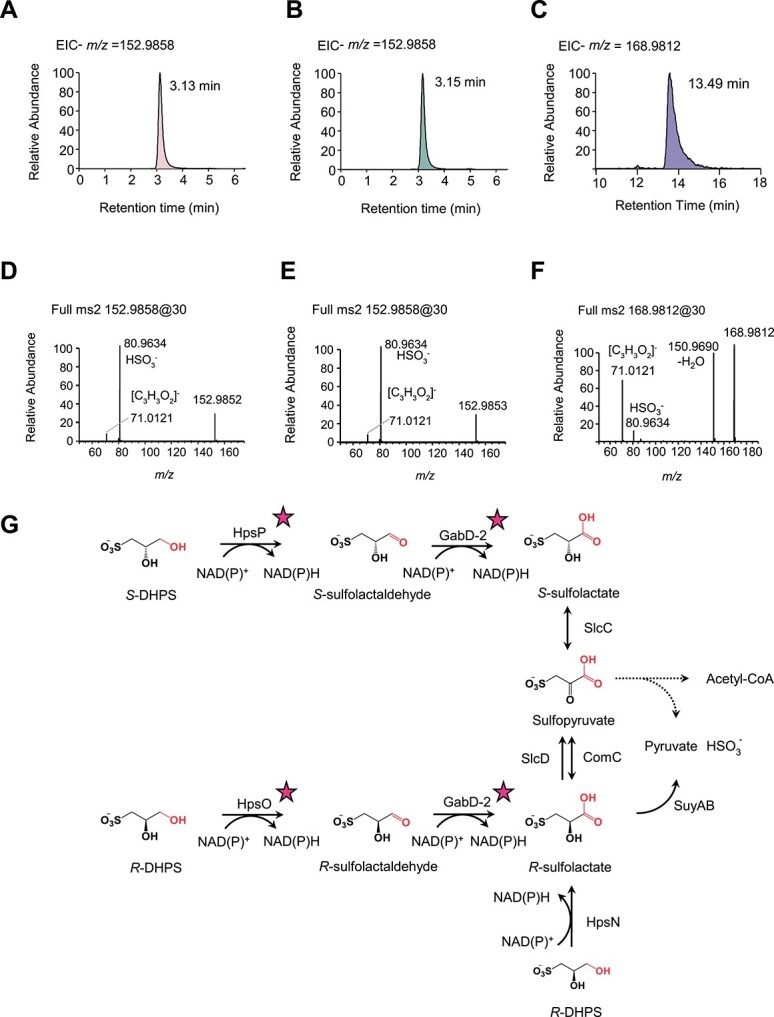
DHPS reaction products and suggested DHPS catabolic pathway. (**A**) Extracted ion chromatogram of *R*-DHPS in reaction buffer addition of *Rp*HpsO to generate sulfolactaldehyde. The peak at 152.9852 *m/z* corresponds to the [M-H]^−^ ions of sulfolactaldehyde (C_3_H_5_O_5_S^−^). (**B**) Extracted ion chromatogram of *S*-DHPS in reaction buffer addition of *Rp*HpsP to generate sulfolactaldehyde. The peak at 152.9853 *m/z* corresponds to the [M-H]^−^ ions of sulfolactaldehyde (C_3_H_5_O_5_S^−^). (**C**) Extracted ion chromatogram of *R*-DHPS in reaction buffer addition of *Rp*HpsN to generate sulfolactate. The peak at 168.9812 *m/z* corresponds to the [M-H]^−^ ions of sulfolactate (C_3_H_5_O_6_S^−^). (**D**) MS/MS fragmentation of sulfolactaldehyde generated in *Rp*HpsO reaction with *R*-DHPS containing HSO_3_^−^ (*m*/*z* 81) and [C_3_H_3_O_2_]^−^ (*m*/*z* 71) ions. (**E**) MS/MS fragmentation of sulfolactaldehyde generated in *Rp*HpsP reaction with *S*-DHPS contains HSO_3_^−^ (81) and [C_3_H_3_O_2_]^−^ (*m*/*z* 71) ions. (**F**) MS/MS fragmentation of sulfolactate generated in *Rp*HpsN reaction with *R*-DHPS lead to loss of a water (−18) and to the formation HSO_3_^−^ (*m*/*z* 81) and [C_3_H_3_O_2_]^−^ (*m*/*z* 71) ions. (**G**) *R*- and *S***-**DHPS catabolic pathway. The enzymes and intermediates are shown, and the reactive groups are colored in red. HpsO, *R*-DHPS dehydrogenase; HpsP, *S*-DHPS dehydrogenase; HpsN, *R*-DHPS dehydrogenase; GabD-2, *R*- and *S*-sulfolactaldehyde dehydrogenase. SlcC, *S*-sulfolactate dehydrogenase; SlcD and ComC, *R*-sulfolactate dehydrogenase. SlcC and ComC convert *S*-sulfolactate and *R*-sulfolactate to sulfopyruvate, respectively [51, 73]. SuyAB, *R*-sulfolactate sulfolyase. Star symbols represent the updated steps in the previously assumed pathway [61]. Dashed lines indicate that the multiple-step reactions involve the conversion of sulfopyruvate to pyruvate, acetyl-CoA, and bisulfite. The representative bacteria of SAR11, *Roseobacteraceae*, and *Burkholderiaceae* contain this suggested DHPS catabolic pathway (Supplementary Data 2).

We investigated the enantioselectivity and biochemical characterization of three proteins from the marine bacterium *R. pomeroyi* DSS-3: HpsO (*Rp*HpsO), HpsP (*Rp*HpsP), and HpsN (*Rp*HpsN). We found that both *Rp*HpsO and *Rp*HpsN specifically bound to *R*-DHPS, catalyzing its reaction with NAD(P)^+^ as cofactors (Fig. 2A-D and Supplementary Table S3). These results indicated that *Rp*HpsO and *Rp*HpsN have enantioselectivity for *R*-DHPS. In contrast, *Rp*HpsP from *R. pomeroyi* DSS-3 catalyzed the reaction of *S*-DHPS and NAD(P)^+^ to form NADH, but did not catalyze *R*-DHPS (Fig. 2E and F and Supplementary Table S3). This demonstrated that *Rp*HpsP is a strict chiral selective enzyme, directly and specifically oxidizing *S*-DHPS. To identify the reaction product, we subjected the reaction mixtures of *Rp*HpsO, *Rp*HpsP, and *Rp*HpsN catalyzing DHPS to HPLC-MS/MS analysis. We detected a novel peak in the catalytic products of the *Rp*HpsO- and *Rp*HpsP-mediated reactions. This peak had a shorter retention time than DHPS and a mass-to-charge ratio (*m/z*) of 152.9858 as a quasi-molecular ion in the negative ion mode ([M-H]^−^) (Fig. 3A and B). The MS/MS fragments of the product were *m/z* 80.9634 and 71.0121 (Fig. 3D and E). These values were identical to the retention time, exact mass, and MS/MS fragment pattern of the sulfolactaldehyde standard (Supplementary Fig. S4A and B). This demonstrated that *Rp*HpsO and *Rp*HpsP catalyzed the conversion of *R*-DHPS and *S*-DHPS to sulfolactaldehyde, respectively. The reaction product of *Rp*HpsN catalyzing *R*-DHPS was identified as sulfolactate by its retention time, exact mass of the [M-H]^−^ ion, and MS/MS fragmentation pattern (Fig. 3C and F and Supplementary Fig. S4C and D). The reactions of *R*-, *S*-DHPS catalyzed by HpsO, HpsP, and HpsN were all transformations of alcohol to aldehyde or carboxylic acid. This indicated that the reaction centers of both *R*- and *S*-DHPS were the C3-OH group (Fig. 3G).

*R. pomeroyi* DSS-3 harbors a gene (*gabD-*2, locus tag: SPO3328) that displayed substantial homology (amino acid sequence identity, 60%) to sulfolactaldehyde dehydrogenase from *Pseudomonas putida* SQ1 (Locus tag: PpSQ1_00088) [50]. We incubated recombinant GabD-2 of *R. pomeroyi* DSS-3 with sulfolactaldehyde and NAD^+^. The accumulation of NADH over time indicated the sulfolactaldehyde was oxidized (Supplementary Fig. S4E). A peak in the reaction mixture had the same retention time, exact mass of the [M-H]^−^ ion and MS/MS fragmentation pattern as the sulfolactate standard (Supplementary Fig. S4F and G), demonstrating the formation of sulfolactate. These results confirmed that GabD-2 is a sulfolactaldehyde dehydrogenase. We also observed that sulfolactate peaks were detected in both mixtures: *Rp*HpsO-mediated *R*-DHPS reaction supplemented with GabD-2 and *Rp*HpsP-mediated *S*-DHPS reaction supplemented with GabD-2 (Supplementary Fig. S4H). However, no sulfolactate formation was observed in the GabD-2 supplemented mixtures of *R*- or *S*-DHPS and NAD^+^. These results suggested that the conjunction of *Rp*HpsO or *Rp*HpsP with GabD-2 converts *R*- or *S*-DHPS to sulfolactate, respectively (Fig. 3G). Moreover, we detected DHPS, sulfolactaldehyde, and sulfolactate among the intracellular metabolites of *R. pomeroyi* DSS-3 and another *Roseobacteraceae* strain, *D. shibae* DFL 12 (Supplementary Figs S5 and S6), when *R*- and *S*-DHPS were individually used as carbon sources for these bacteria. Thus, the formation of sulfolactaldehyde from *R*-DHPS and *S*-DHPS by HpsO and HpsP, respectively, and the subsequent formation of sulfolactate by GabD-2 (Fig. 3G), expands known bacterial sulfonate metabolic pathways [51].

### Enantioselective transformation of DHPS by HpsO, HpsP, and HpsN in *Roseobacteraceae* strains

**Figure 4:**
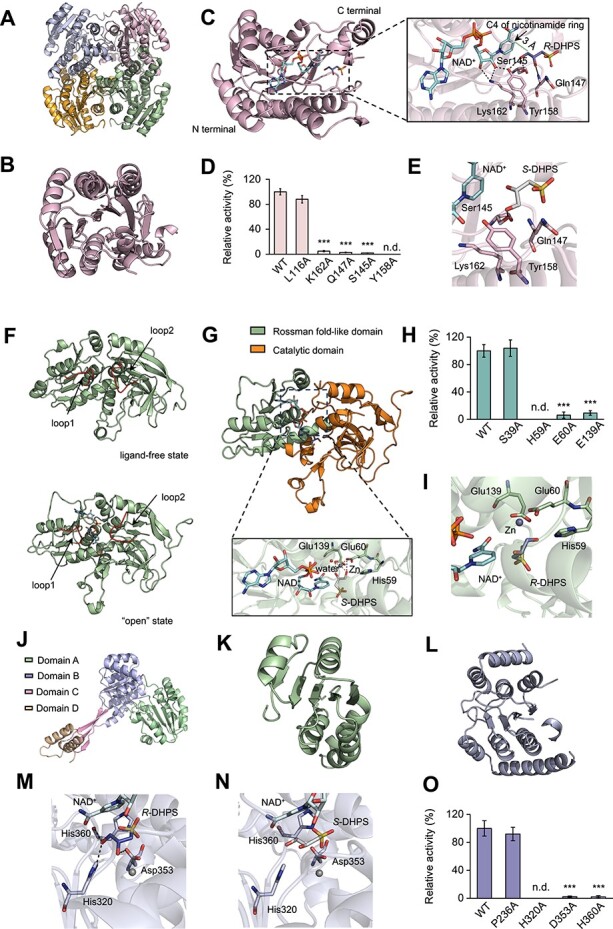
Structural insights of *Rp*HpsO. (**A**) Overall structure of the *Rp*HpsO tetramer in the asymmetric unit. The tetramer is composed of two dimers, each of which is formed by two identical monomers. (**B**) Cartoon diagram of the *Rp*HpsO monomer with Rossmann fold. (**C**) the structural details of *Rp*HpsO with NAD^+^ and *R*-DHPS after molecular dynamics simulation. Active site residues Ser145, Gln147, Tyr158, and Lys162 are shown as sticks. (**D**) the relative enzymatic activities of *Rp*HpsO mutants toward *R*-DHPS. The activity of wild type (WT) *Rp*HpsO toward *R*-DHPS was used as reference (100%). (**E**) The structure of *Rp*HpsO docked with NAD^+^ and *S*-DHPS. (**F**) The structure of *Ds*HpsP monomer in a ligand-free state and ligand-binding state after docking and molecular dynamics simulation. Loop1 (Gly249-Gly253) and loop2 (Asp46-Ala53) are color in red.(**G**) the structure of *Ds*HpsP monomer with NAD^+^ and *S*-DHPS after molecular dynamics simulation. The monomer consists of two domains, the Rossman fold-like domain (Leu132-Ile270) colored in green and a catalytic domain (Met1-Pro131, Gly271-Pro327) colored in orange. The active sites are located in the cleft between these two domains. Active site residues (His59, Glu60, and Glu139) are shown as sticks. (**H**) The relative enzymatic activities of *Ds*HpsP mutants toward *S*-DHPS. The activity of WT *Ds*HpsP toward *S*-DHPS was used as reference (100%). (**I**) The structure of *Ds*HpsP docked with NAD^+^ and *R*-DHPS. (**J**) The structure of the optimized *Rp*HpsN monomer contains four domains (individually colored). Domain A (green), Val218-Leu374; domain B (purple), Ser18-Leu93, Val113-Arg217, Ser375-Tyr379; domain C (pink), Thr94-Pro112, Met380-Tyr393; domain D (orange), Lys394-Leu406. (**K**) Domain A contains five-stranded parallel β-sheets, sandwiched between five α-helices, forming a Rossmann-like fold. (**L**) Domain B folds into Rossmann-like fold at the core segment surrounded by two V-shaped α-helices. (**M**) The structural details of *Rp*HpsN with NAD^+^ and *R*-DHPS after molecular dynamics simulation. Active site residues His320, Asp353, and His360 are shown as sticks. Metal atom (grey) represents the predicted site of Zn^2+^. (**N**) The structure of *Rp*HpsN docked with NAD^+^ and *S*-DHPS. (**O**) the relative enzymatic activities of *Rp*HpsN mutants toward *R*-DHPS. The activity of WT toward *R*-DHPS was used as reference (100%). Black dished lines represent hydrogen bonds. Green dashed line represents the distance between atoms. Orange dashed lines represent the coordinate bonds. ^*^^*^^*^, *P* < .001 (*t*-test). n.d., not detectable.

The crystal structure of *Rp*HpsO shows that it exists as a tetramer in the asymmetric unit (Fig. 4A), with seven-stranded parallel β-sheets sandwiched by six α-helices, constituting a typical Rossmann-fold domain [52] in each monomer (Fig. 4B). *Rp*HpsO is a member of the short chain dehydrogenase/reductase (SDR) family [53], which has two defining features, an N-terminal domain responsible for coenzyme NAD(H) or NADP(H) binding and a C-terminal domain harboring the essential catalytic residues [54]. We docked the molecules of NAD^+^ and *R*-DHPS into the structure of *Rp*HpsO and conducted a molecular dynamics simulation. The results showed that the orientation of the C3-OH of *R*-DHPS was toward the reactive center (C4) of the nicotinamide ring of NAD^+^ with a distance of ~3 Å, which was appropriate for hydride transfer (Fig. 4C). Ser145 and Tyr158 both formed hydrogen-bonding interactions with the C3-OH of *R*-DHPS (Fig. 4C). The ribose, adjacent to nicotinamide of NAD^+^, was observed to form hydrogen bonds with Tyr158 and Lys162 (Fig. 4C). The Ser, Tyr, and Lys residues are conserved in other SDR family enzymes and constitute a catalytic triad [53–55], suggesting their essential role in *Rp*HpsO catalyzation of *R*-DHPS. *Rp*HpsO mutants of Ser145Ala, Tyr158Ala, and Lys162Ala were obtained and showed large drops in enzymatic activities for *R*-DHPS (Fig. 4D). However, enzymatic activity of the Leu116Ala mutant for *R*-DHPS did not decline significantly (Fig. 4D). This demonstrated that Ser145, Tyr158, and Lys162 function as catalytic residues in *Rp*HpsO. Gln147 formed a hydrogen bond with the chirality-center hydroxy (C2-OH) of *R*-DHPS, suggesting that Gln147 may have a vital role in specific recognition of *R*-DHPS. The hydrogen-binding interaction between Gln147 and DHPS disappeared when *R*-DHPS was replaced with *S*-DHPS in the *Rp*HpsO structure (Fig. 4E). Mutant Gln147Ala abolished the most enzymatic activity of *Rp*HpsO for *R*-DHPS (Fig. 4D). These results demonstrated that the interaction between Gln147 of *Rp*HpsO and C2-OH of *R*-DHPS appears to drive the specificity of *Rp*HpsO for the *R*-DHPS enantiomer.

HpsP have a strict stereoselectivity for *S*-DHPS via the interactions with the C2-OH of *S*-DHPS. We solved the structure of *Ds*HpsP from *D. shibae* DFL 12, which shared a substantial amino acid sequence identity (68%) with *Rp*HpsP from *R. pomeroyi* DSS-3 and also possesses enantioselectivity for *S*-DHPS (Supplementary Fig. S7A). The crystal structure of *Ds*HpsP showed that four monomers, each containing two Zn^2+^ ions, constituted a tetramer in the asymmetric unit. *Ds*HpsP is a member of the medium-chain alcohol dehydrogenases/reductase (MDR) superfamily, with a Rossmann-like fold domain for co-factor binding and a catalytic domain harboring essential catalytic residues and a metal atom. The docked *Ds*HpsP complex model containing *S*-DHPS and NAD^+^ was subjected to molecular dynamics simulation analysis. The results showed that two loops located at the entrance of the ligand binding pocket underwent an apparent conformational change. Loop 1 and loop 2 exhibited a rather constricted channel in the ligand-free state (Fig. 4F), but displayed an “open” state, with movement apart, upon binding of NAD^+^ and *S*-DHPS (Fig. 4F), suggesting a gating function for these loops in ligand entry. In the “open” state, the C3-OH of *S*-DHPS formed hydrogen-bonding interactions with His59, Glu60 and Glu139, as well as coordinate bonds with Zn^2+^ (Fig. 4G). The enzymatic activity of *Ds*HpsP for *S*-DHPS was nearly abolished after removal of metals by 1, 10-phenanthroline. The addition of ZnCl_2_ and MnCl_2_ to metal-chelated *Ds*HpsP could restored enzymatic activity toward *S*-DHPS (Supplementary Fig. S7B). ICP-MS analysis revealed the presence of Zn^2+^ and Mn^2+^ in the protein (Supplementary Table S4). These results indicated that metals play an essential role in *Ds*HpsP’s catalytic function. Site-directed mutagenesis suggested that His59 functions as the catalytic residue, as mutant His59Ala completely abolished the catalytic activity for *S*-DHPS (Fig. 4H). Glu60Ala and Glu139Ala displayed weak catalytic activities for *S*-DHPS, implying that Glu60 and Glu139 probably play roles in binding *S*-DHPS (Fig. 4H). The chirality-center C2-OH of *S*-DHPS could form a hydrogen bond with Glu139 and a coordinate bond with Zn^2+^ (Fig. 4G), and these interactions disappeared when *R*-DHPS was replaced with *S*-DHPS at the active pocket, due to steric hindrance caused by the reverse chiral direction of C2-OH in *R*-DHPS (Fig. 4I). Moreover, C2-OH of *R*-DHPS could not form stable interactions with other surrounding residues (Fig. 4I). The overall structure of *Ds*HpsP was similar to AlphaFold2-predicted *Rp*HpsP structures from *R. pomeroyi* DSS-3 with a RMSD value of 0.83 Å over 325 residues (Supplementary Fig. S7A). Moreover, the essential residues His59, Glu60, and Glu139 of *Ds*HpsP from *D. shibae* DFL 12 were conserved in *Rp*HpsP (Supplementary Fig. S7A). Together, these suggest their common enzymatic functions and mechanisms, in which the interactions with C2-OH of *S*-DHPS determine the stereoselectivity of HpsP.

The *Rp*HpsN structure consists of four molecules arranged as a tetramer in an asymmetric unit. Each *Rp*HpsN monomer contains two smaller domains (domains: C and D) and two large domains (domains: A and B) harboring Rossmann-like folds (Fig. 4J-L). Sequence-based functional prediction suggested that *Rp*HpsN belongs to the aldehyde dehydrogenase (ALDH) superfamily and is a metalloenzyme containing a Zn^2+^-binding region. Although the electron density indicative of Zn^2+^ was not observed in protein structure of *Rp*HpsN, ICP-MS analysis confirmed the presence of Zn^2+^ and Mn^2+^ in the protein (Supplementary Table S4). The enzymatic activity of *Rp*HpsN toward *R*-DHPS was completely abolished after metals removal by EDTA. *Rp*HpsN recovered 60% and 88% of native enzymatic activities after addition of Zn^2+^ and Mn^2+^ to metal-chelated reaction mixtures, respectively (Supplementary Fig. S7B), demonstrating that *Rp*HpsN is a metal-dependent enzyme. A docked complex model comprised of Zn^2+^-containing *Rp*HpsN and *R*-DHPS with NAD^+^ was generated and further optimized by molecular dynamics simulation. The final simulation results showed that *R*-DHPS was in close proximity to the nicotinamide of NAD^+^ (Fig. 4M). The C3-OH of *R*-DHPS could form hydrogen bond interactions with NE2 of His320 and O of His360, respectively (Fig. 4M). In the hydrogen bond interaction between C3-OH of *R*-DHPS and NE2 of His320, 3-OH of *R*-DHPS was the hydrogen donor and NE2 of His320 was the hydrogen acceptor, suggesting that H^+^ from the former was abstracted by the latter. Residue His320 from *Rp*HpsN is conserved in l-histidinol dehydrogenase (His327) from *E. coli* MC1061, where it functions as a general base participating in catalysis [36], implying a potentially similar function for His320 in *Rp*HpsN. Mutagenesis His320Ala of *Rp*HpsN was conducted and completely abolished enzymatic activity toward *R*-DHPS (Fig. 4O), indicating the essential catalytical function of His320 for *R*-DHPS catalyzation. The chirality-center C2-OH of *R*-DHPS, oriented toward reverse direction of NAD^+^ and formed a hydrogen bond with OD2 of Asp353 (Fig. 4M). When residue Asp353 was substituted with Ala, the enzymatic activity for *R*-DHPS declined significantly (*t*-test, *P* < 0.001) (Fig. 4O), indicating that Aps353 played an important role in *Rp*HpsN binding of *R*-DHPS. Furthermore, when *R*-DHPS was replaced with *S*-DHPS in *Rp*HpsN complex model, the hydrogen bond could not be formed between C2-OH of *S*-DHPS and surrounding residues of *Rp*HpsN (Fig. 4N). Together, this suggested that the interaction between chiral group C2-OH of *R*-DHPS and Asp353 determined *Rp*HpsN enantioselectivity.

### Distribution of DHPS dehydrogenase genes in diverse bacteria and the *Tara* oceans

**Figure 5:**
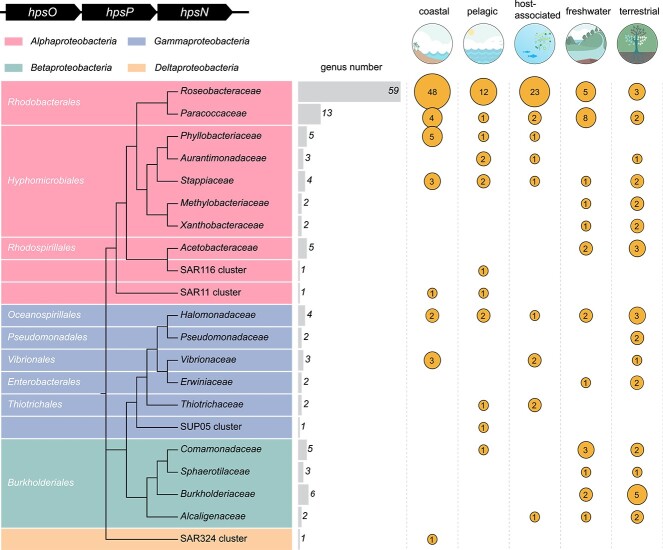
Distribution of homologs of *R-* and *S*-DHPS dehydrogenase genes. Maximum likelihood phylogenetic tree of bacteria containing candidate genes encoding *hpsO*, *hpsP*, and *hpsN* in an operon, and SAR11. The habitats of these genomes were classified as coastal, pelagic, host-associated, freshwater, and terrestrial environments. The size of the circles and figures within them indicate the number of genera isolated from the corresponding environments. Empty space indicates no genera were found in the respective environment.

**Figure 6:**
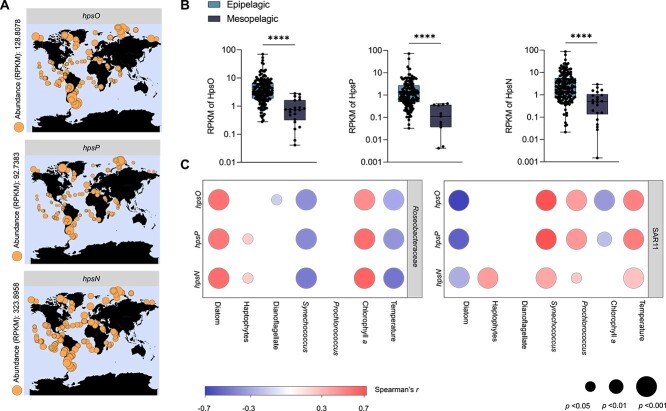
**Distribution of homologs of *R-* and *S*-DHPS dehydrogenase genes in *Tara* oceans.** (**A**) Distribution of *hpsO*, *hpsP*, and *hpsN* in the epipelagic ocean (<200 m). The circle size indicates the relative transcripts abundance of genes. (**B**) The transcript abundances of *hpsO*, *hpsP*, and *hpsN* from *Roseobacteraceae* in the epipelagic ocean (<200 m) and mesopelagic ocean (200–1000 m). ^*^^*^^*^^*^, *P* < .0001 (Mann Whitney test). (**C**) The correlation analysis among gene expression from *Roseobacteraceae* and SAR11 with environmental factors and phytoplankton. Color represents Spearman’s *r*. circle size represents statistical significance. Empty space indicates no significant correlation values (*P* ≥ .05).

A total of 609 bacterial genomes, spanning 126 genera, contained candidate *hpsO*, *hpsP*, and *hpsN* (Fig. 5 and Supplementary Data 3). The most prevalent taxon was *Roseobacteraceae*, accounting for 59/126 of potential DHPS-utilizing bacterial genera. These bacteria were isolated from a variety of environments, including coastal, pelagic, host-associated, freshwater, and terrestrial environments (Fig. 5), suggesting that the bacterial metabolism for chiral DHPS is widespread in these environments. More than half of these genera (67/126) were isolated from eutrophic coastal regions, with only a small number of bacteria from deep-sea environments (Supplementary Data 3). *Tara* Oceans data showed that the abundance of *hpsO*, *hpsP*, and *hpsN* transcripts from *Roseobacteraceae* were significantly higher (Mann Whitney test, *P* < 0.0001) in the epipelagic zone (depth, <200 m) than the mesopelagic zone (depth, 200–1000 m) in the global ocean (Fig. 6A and B), suggesting that *hpsO*, *hpsP*, and *hpsN* were primarily expressed in the sunlit epipelagic ocean where phytoplankton thrive. *Roseobacteraceae* and SAR11 were the two largest contributors to the transcripts of *hpsO*, *hpsP*, and *hpsN* at most *Tara* Oceans stations, accounting for >50% of these DHPS dehydrogenase genes transcripts in total (Supplementary Data 4).

## Discussion

This study provides evidence of the existence of both enantiomers of a chiral substance in marine phytoplankton. The biosynthesis of DHPS in phytoplankton is not fully understood, but it is thought to proceed through the reduction of cysteinolic acid to form the ketone compound 2-oxo-3-hydroxy-propane-1-sulfonate, a prochiral precursor to DHPS [51]. Both cysteinolic acid and 2-oxo-3-hydroxy-propane-1-sulfonate were detected in all DHPS-producing diatoms and coccolithophores (Supplementary Figs S8-S10). To our knowledge, ketoreductases can exhibit ambiguous or high stereoselectivity to produce chiral alcohols, depending on the orientations of the substrate and coenzyme in the enzyme’s active pocket [56–59]. Therefore, the stereoselectivity of the ketoreductase that catalyzes the reduction of the prochiral ketone to form DHPS may be a key determinant of the chirality of DHPS produced in phytoplankton, and requires further study.

DHPS have two hydroxyl groups probably serving as reactive handles for enzymatic reactions, which could provide potential pathways for DHPS degradation in metabolically diverse microbe [60]. In this novel DHPS catabolism pathway, the primary hydroxyls of *R*-DHPS and *S*-DHPS are directly oxidized by HpsO and HpsP, respectively, without requiring *S*-DHPS racemization, as previously suggested in soil bacteria *Cupriavidus pinatubonensis* JMP134 [61]. HpsO (*Cp*HpsO, locus tag: Reut_C6091), HpsP (*Cp*HpsP, Reut_C6089), and HpsN (*Cp*HpsN, Reut_C6092) from *C. pinatubonensis* JMP134 exhibited the same chiral selectivity for DHPS as *Roseobacteraceae* strains (Supplementary Fig. S11A, C and E). This is similar to the strategy used by marine bacteria, which mainly utilize d-amino acids as a carbon source by oxidative deamination with d-amino acid oxidases, rather than racemizing them into l-amino acids [62]. *Roseobacteraceae* are shown to be the main contributors to the transformation of DHPS in the coastal waters of China [20]. On a global scale, the abundance of *hpsO*, *hpsP*, and *hpsN* transcripts from *Roseobacteraceae* strongly correlates with diatoms and chlorophyll *a* (Fig. 6C and Supplementary Table S5). SAR11, another prevalent marine taxon, has been shown to be able to catabolize DHPS [51]. We further confirmed that the homologs of HpsO (*Cu*HpsO, PU1002_02121), HpsP (*As*HpsP, HIMB5b_00005240), and HpsN (*Cu*HpsN, PU1002_02401) from SAR11 exhibited the same substrate specificity for chiral DHPS as *Roseobacteraceae* (Supplementary Fig. S11B, D and F). In the pelagic surface ocean, SAR11 bacteria had a much higher abundance of *hpsP* and *hpsN* transcripts than *Roseobacteraceae* (Supplementary Data 4). For example, in the North Pacific Subtropical Gyre, the predominant SAR11 clade was the source for the majority of transcripts associated with DHPS catabolism, with a lower abundance for the *Roseobacteraceae* family [51]. These findings suggest SAR11 plays a major role in DHPS transformation in oligotrophic marine environments. Therefore, *Roseobacteraceae* and SAR11 appear to be the primary degraders of DHPS in seawater through DHPS dehydrogenases with enantioselective substrate specificity.

Within the heterogeneous SDR, MDR, and ALDH superfamilies, HpsO, HpsP, and HpsN are most structurally similar to d-gluconate dehydrogenase, d-sorbitol dehydrogenase, and l-histidinol dehydrogenase, respectively. Despite their low sequence identity (<40%) to their homologs, these enzymes shared the ancient Rossmann-fold domain for dinucleotide-binding motif and conserved catalytic sites (Supplementary Fig. S12). However, substantial amino acid substitutions around the substrate channel, catalytic center, and protein surface have resulted in significant differences in the electrostatic potential energy between *Rp*HpsO, *Ds*HpsP, and *Rp*HpsN compared with their homologs (Supplementary Fig. S13), which likely lead to changes in intramolecular networks, thereby contributing to functional specialization [18]. Specifically, the severely non-conserved regions of *Rp*HpsO (Arg192-Ala201), the variation of residues constituting gate-function loops in *Ds*HpsP’s substrate channel created significant confirmational changes, and the triple substitution of *Rp*HpsN (Arg124, Tyr125, and Ser126) surrounding the catalytic center, (Supplementary Fig. S14), probably perturbing the electrostatics of the catalytic center and fine tune the enzymatic local dynamics. Indeed, we observed *Rp*HpsO, *Ds*HpsP, and *Rp*HpsN exhibited enzymatic activity for the oxidation of d-gluconate, d-sorbitol, and l-histidinol at lower catalytic efficiency (*k*_cat_/*K_m_*) (3460-, 52-, and 691-fold, respectively) than their DHPS activities (Table 1 and Supplementary Fig. S15). Interestingly, d-gluconate dehydrogenase, d-sorbitol dehydrogenase, and l-histidinol dehydrogenase also exhibit enzymatic activities for DHPS, with comparable catalytic efficiencies to their canonical activities (Table 1 and Supplementary Fig. S15). However, their affinities for DHPS (1/*K_m_*) are much lower (up to 76-fold) than that of *Rp*HpsO, *Ds*HpsP, and *Rp*HpsN, respectively (Table 1 and Supplementary Fig. S15). The binding residue Gln147 in *Rp*HpsO is not conserved in d-gluconate dehydrogenase. His69 is a binding site in d-sorbitol dehydrogenase [63], while the corresponding residue (His59) is the catalytic residue in *Ds*HpsP (Fig. 4G and H), suggesting the different substrates induce shifts in the roles of active sites [64]. In *Roseobacteraceae* genomes, *hpsO*, *hpsP*, and *hpsN* are often found in an operon together with DHPS transporters and a transcriptional regulator. These genes are co-transcribed and co-expressed in the presence of *R*- or *S*-DHPS [20]. Biochemical and genetic evidence suggest that HpsO, HpsP, and HpsN from *Roseobacteraceae* specialize in DHPS catalysis. In contrast, the homologs of *hpsO*, *hpsP*, and *hpsN* in the SAR11 genome are not clustered in a single operon. They exhibit low to moderate sequence identities to their *Roseobacteraceae* counterparts (Supplementary Data 3), as well as distinct electrostatic potential energies (Supplementary Fig. S13). Their activities for DHPS and the secondary substrates were comparable (Table 1 and Supplementary Fig. S15). This provides a valuable example of promiscuous activities in SAR11 with a streamlined genome, allowing them to scavenge more substrates for survival.

**Table 1:** Michaelis–Menten kinetics of DHPS dehydrogenases.

		Substrates	*k*_cat_ (s^−1^)	K*_m_* (mM)	*k*_cat_/K*_m_* (mM^−1^ s^−1^)	Catalytic efficiency ratio	1/K*_m_* (mM)	Ratio of affinity for DHPS
*Roseobacteraceae*	*Rp*HpsO	*R*-DHPS	0.023 ± 0.002	0.85 ± 0.24	0.027 ± 0.002	3460.4	1.18 ± 0.07	43.9
		d-gluconate	0.002 ± 0.001	161.19 ± 65.31	8.0 × 10^−6^ ± 4.0 × 10^−6^		6.2 × 10^−3^ ± 5.0 × 10^−3^	
	*Ds*HpsP	*S*-DHPS	0.183 ± 0.021	26.02 ± 9.20	0.007 ± 0.001	51.6	0.04 ± 0.004	3.6
		d-sorbitol	0.026 ± 0.010	189.23 ± 143.25	1.4 × 10^−4^ ± 5.0 × 10^−5^		5.3 × 10^−3^ ± 9.6 × 10^−4^	
	*Rp*HpsN	*R*-DHPS	0.059 ± 0.001	1.07 ± 0.83	0.055 ± 0.001	691.3	0.74 ± 0.45	76.3
		l-histidinol	0.012 ± 0.005	154.95 ± 62.71	8.0 × 10^−5^ ± 4.0 × 10^−5^		6.5 × 10^−3^ ± 1.1 × 10^−4^	
SAR11	*Cu*HpsO	*R*-DHPS	0.021 ± 0.005	103.10 ± 47.34	2.1 × 10^−4^ ± 5.1 × 10^−5^	0.7	9.7 × 10^−3^ ± 3.1 × 10^−3^	
		d-gluconate	0.016 ± 0.003	53.50 ± 28.53	2.9 × 10^−4^ ± 6.3 × 10^−5^		0.02 ± 0.002	
	*As*HpsP	*S*-DHPS	0.211 ± 0.007	64.35 ± 11.27	3.0 × 10^−3^ ± 1.0 × 10^−4^	25.0	0.02 ± 0.002	
		d-sorbitol	0.021 ± 0.007	163.81 ± 67.65	1.3 × 10^−4^ ± 4.5 × 10^−5^		6.1 × 10^−3^ ± 5.6 × 10^−4^	
	*Cu*HpsN	*R*-DHPS	0.179 ± 0.025	106.32 ± 48.97	1.7 × 10^−3^ ± 2.3 × 10^−4^	2.2	0.015 ± 0.006	
		l-histidinol	0.012 ± 0.001	16.12 ± 2.59	7.5 × 10^−4^ ± 4.3 × 10^−5^		0.04 ± 0.005	
Other species	d-gluconate dehydrogenase	*R*-DHPS	0.009 ± 0.001	37.14 ± 2.85	2.5 × 10^−4^ ± 3.2 × 10^−5^	0.2	0.03 ± 0.006	
		d-gluconate	0.014 ± 0.005	12.96 ± 6.74	1.1 × 10^−3^ ± 4.0 × 10^−4^		0.08 ± 0.02	
	d-sorbitol dehydrogenase	*S*-DHPS	0.028 ± 0.001	94.16 ± 4.68	2.9 × 10^−4^ ± 1.2 × 10^−5^	0.4	0.01 ± 0.003	
		d-sorbitol	0.027 ± 0.001	32.35 ± 3.59	8.3 × 10^−4^ ± 4.5 × 10^−5^		0.04 ± 0.02	
	l-histidinol dehydrogenase	*R*-DHPS	0.020 ± 0.002	103.10 ± 8.50	1.9 × 10^−4^ ± 1.5 × 10^−5^	0.04	9.7 × 10^−3^ ± 1.8 × 10^−4^	
		l-histidinol	0.042 ± 0.022	9.37 ± 3.63	0.004 ± 0.002		0.11 ± 0.002	

*Rp*HpsO (1.7 μM), *Ds*HpsP (0.4 μM), and *Rp*HpsN (0.6 μM) from *Roseobacteraceae Ruegeria pomeroyi* DSS-3 and *Dinoroseobacter shibae* DFL 12. *Cu*HpsO (0.2 μM), *As*HpsP (0.5 μM), and *Cu*HpsN (0.2 μM) from SAR11 species *Candidatus* Pelagibacter *ubique* HTCC1002 and *Alphaproteobacteria* sp. SAR11 HIMB5. d-gluconate dehydrogenase (0.4 μM) from *Streptococcus suis* sv. 2 BM411a (Locus tag: Ga0130485_10764), d-sorbitol dehydrogenase (0.12 μM) from *Bacillus subtilis* 168 (Bsubs1_010100003458), and l-histidinol dehydrogenase (0.8 μM) from *E. coli* MC1061 (Ga0125172_11). Catalytic efficiency ratio, *k*_cat_/K*_m_* of enzymes for DHPS divided by that of enzymes for other substrates. Ratio of affinity for DHPS, 1/K*_m_* of DHPS dehydrogenases from *Roseobacteraceae* for DHPS divided by that of enzymes from other species for DHPS.

The promiscuous activities of DHPS dehydrogenases in *Roseobacteraceae* may be remnants of an evolutionary ancestor shared by them and their homologs. Promiscuous enzymes from SDRs, MDRs, and ALDHs are regularly used for present-day synthesis of chiral alcohols through directed laboratory evolution and protein engineering techniques [65]. For example, mutations in alcohol dehydrogenase and ketoreductases can confer the ability to produce single enantiomer compounds [56, 57]. Moreover, during short-term evolution of bacteria adapted to grow on non-native chiral carbon sources such as d-arabinose and d-xylulose, enzymes with promiscuous activity were recruited to metabolize enantiomeric compounds via mutation events [66]. These findings suggest that promiscuous activity can be the starting point for the evolution of enantioselectivity in enzyme superfamilies. *Roseobacteraceae* once underwent a genome expansion coinciding with the radiation event of red-lineage algae (coccolithophores, dinoflagellates, and diatoms) in the Mesozoic Era [67, 68], possibly acquiring a large repertoire of genes for metabolizing abundant algal metabolites, including DMSP and DHPS [51]. Based on the structural characterizations and canonical activities of DHPS dehydrogenases and their homologs, the most parsimonious scenario is that their ancestors were promiscuous enzymes with latent functions. After gene duplication events and mutations, enzymes evolved specialization via alterations to enzyme dynamics without directly affecting the conserved catalytic residues.

Bacteria, once widely believed to be unable to metabolize d-tartaric acid, the first mirror-image molecule discovered by Louis Pasteur, and l-glucose, a non-naturally occurring sugar, have been shown to decompose both them [69, 70]. Ocean harbors a remarkable diversity of microbial enzymes, with over 410 000 gene clusters exhibiting exceptional functional breadth and versatility [6]. Marine bacteria can enantioselectively degrade human-made chiral pollutants, such as pharmaceuticals and pesticides, in the environment [71, 72]. This study suggests that enzymatic promiscuity may play a crucial role in enabling bacteria to metabolize heterochiral DHPS. These findings collectively support the hypothesis that microbes possess an extraordinary capacity to cope with the chiral diversity of organic matter in the ocean. In summary, this study uncovers the incorporation of both DHPS enantiomers into the phytoplankton-bacteria metabolic network, as evidenced by their chiral configuration and enantioselective transformation by the stereo-specificity of enzymes, illuminating a previously hidden dimension of metabolic currencies in the marine ecosystem.

## Supplementary Material

Supplementary_Information_wrae084

Supplementary_Data_1_wrae084

Supplementary_Data_2_wrae084

Supplementary_Data_3_wrae084

Supplementary_Data_4_wrae084

## Data Availability

All data are available in the main text or the supplementary materials. The protein structures of *Rp*HpsO, *Ds*HpsP, and *Rp*HpsN were deposited in Protein Data Bank database (https://www.rcsb.org/) under access code 8WWF, 8WWD, and 8WWE, respectively.
