# Supplementary material for: Enantioselective transformation of phytoplankton-derived dihydroxypropanesulfonate by marine bacteria
Source: ISME J. 2024 May 6;18(1):wrae084. doi: 10.1093/ismejo/wrae084 (PMC11131964; doi:10.1093/ismejo/wrae084)
Supplement: Supplementary_Information_wrae084 [file supplementary_information_wrae084.pdf]

Supplementary Information for

Enantioselective transformation of phytoplankton-derived dihydroxypropanesulfonate by marine bacteria

Le Liu[1], Xiang Gao[2], Changjie Dong[1], Huanyu Wang[1], Xiaofeng Chen[3], Xiaoyi Ma[1], Shujing Liu[1], Quanrui Chen[1], Dan Lin[1], Nianzhi Jiao[1], Kai Tang[1]*.

Author for correspondence:

Kai Tang: tangkai@xmu.edu.cn

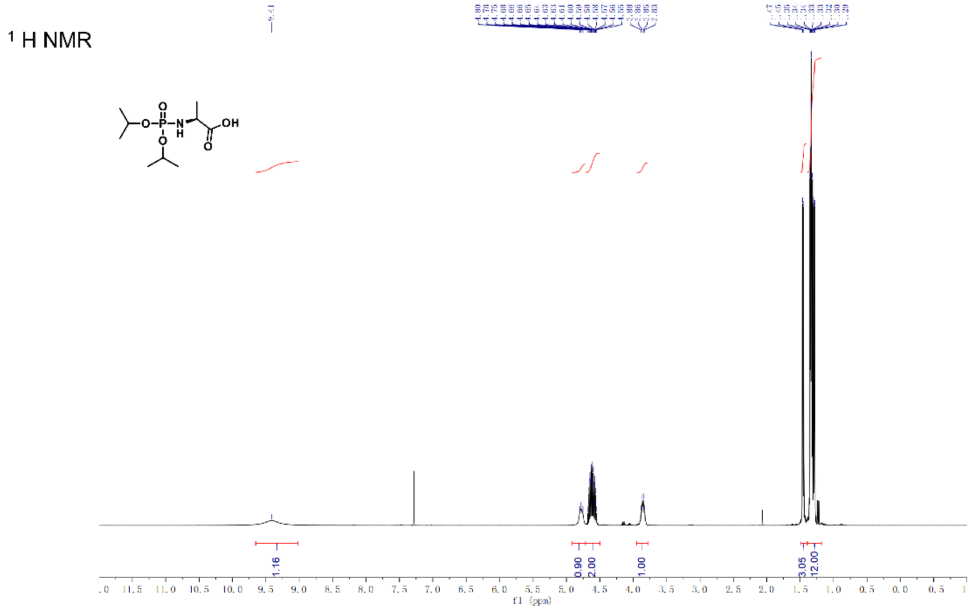

Fig. S1 ^{1}H NMR of synthetic P-L-Ala.

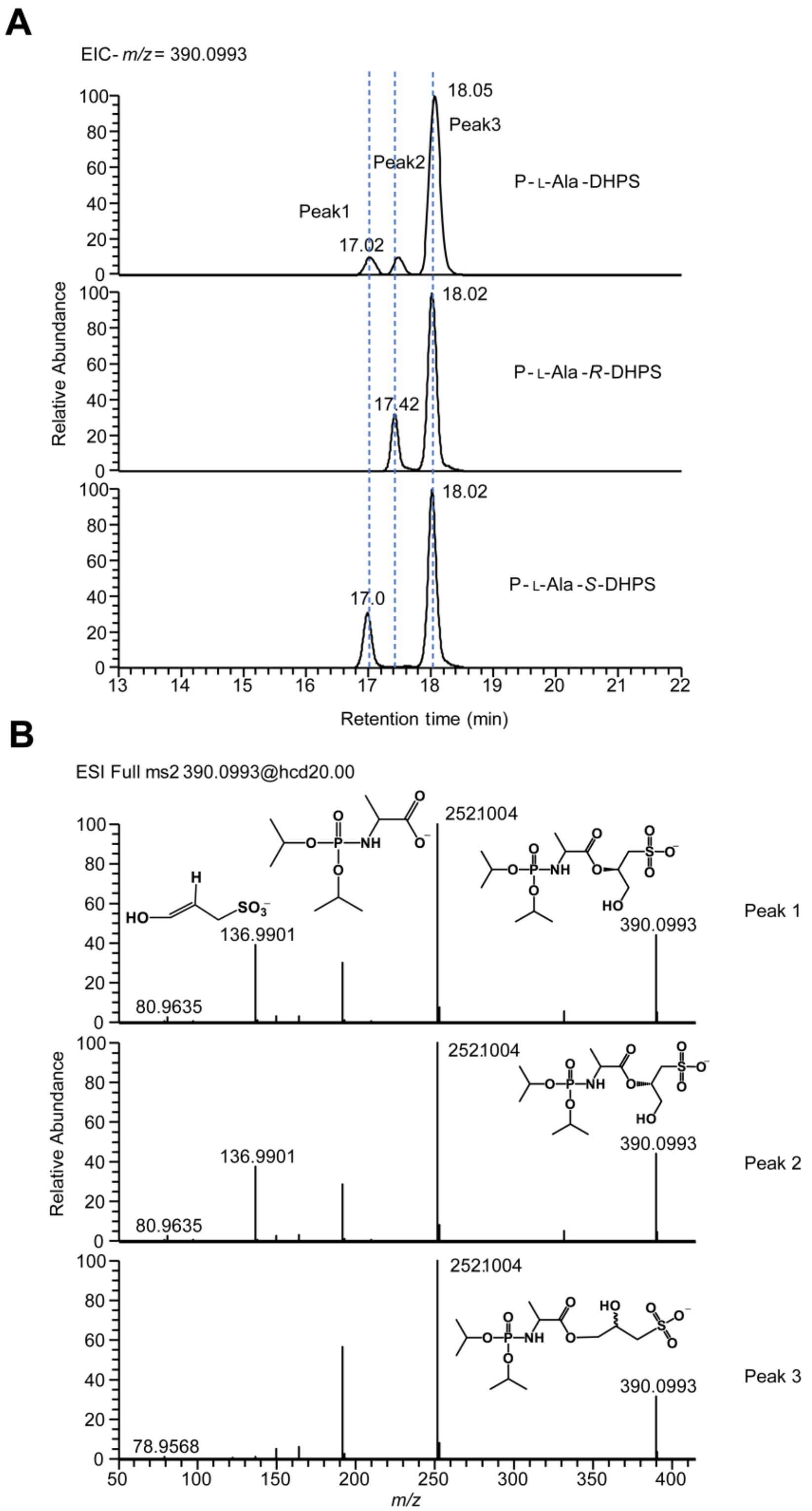

Fig. S2 Fig. S2 LC-MS extracted ion chromatograph of synthetic labeled standard DHPS (mole ratio of *R*:*S* = 1:1) by P-L-Ala (A) and the corresponding MS/MS fragmentation of P-L-Ala-DHPS (B).

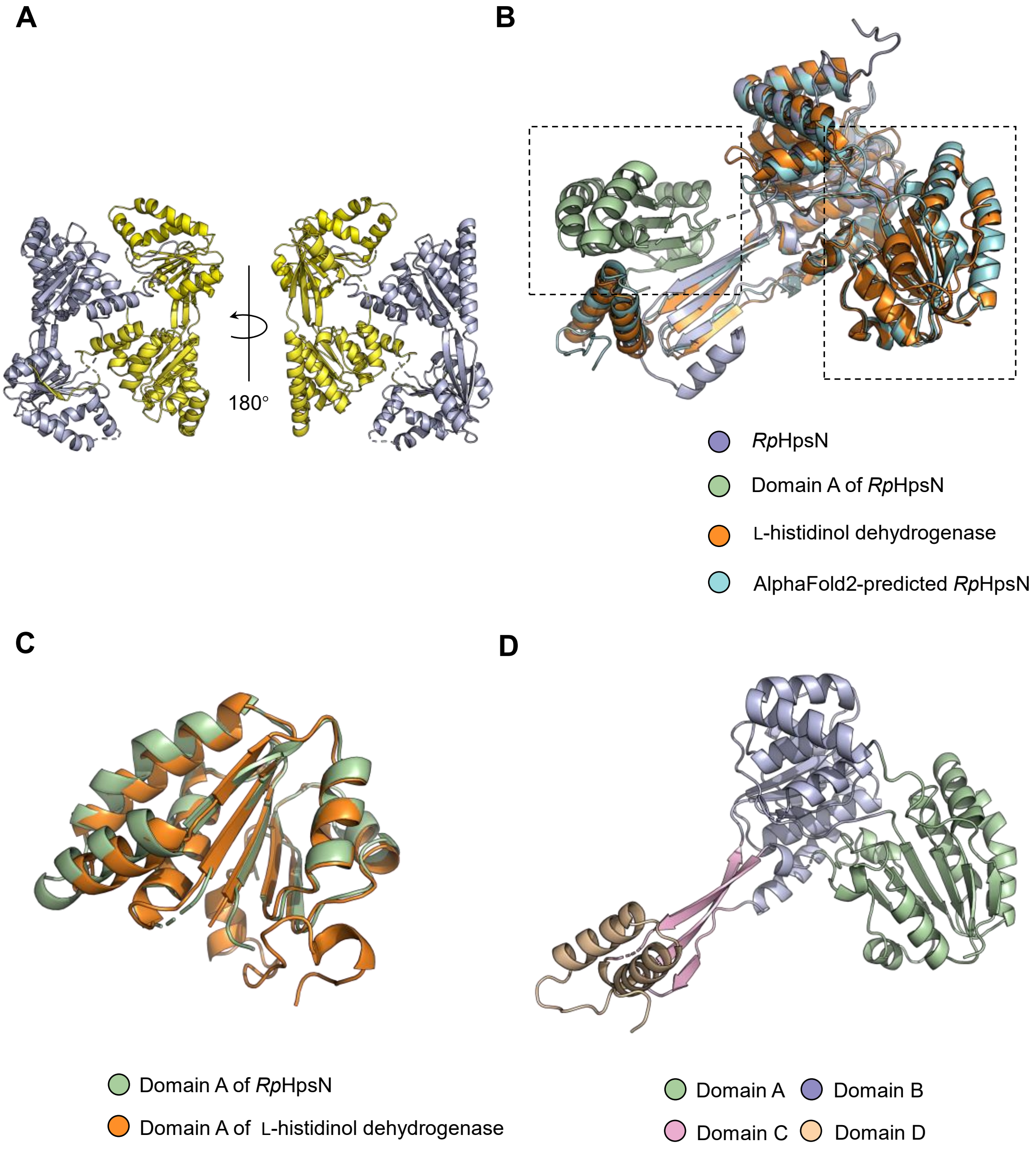

Fig. S3 The optimization of *Rp*HpsN structure. (**A**) Dimer structure of *Rp*HpsN before optimization. (**B**) Structural alignments of the *Rp*HpsN crystal structure with AlphaFold2-predicted *Rp*HpsN and L-histidinol dehydrogenase from *E. coli* MC1061 (PDB ID: 1KAE). The significant difference in spatial position of domain A is shown in dotted boxes. (**C**) Structural alignments of domain A of the *Rp*HpsN crystal structure with domain A of L-histidinol dehydrogenase. (**D**) *Rp*HpsN structure after optimization using AlphaFold2-predicted structure.

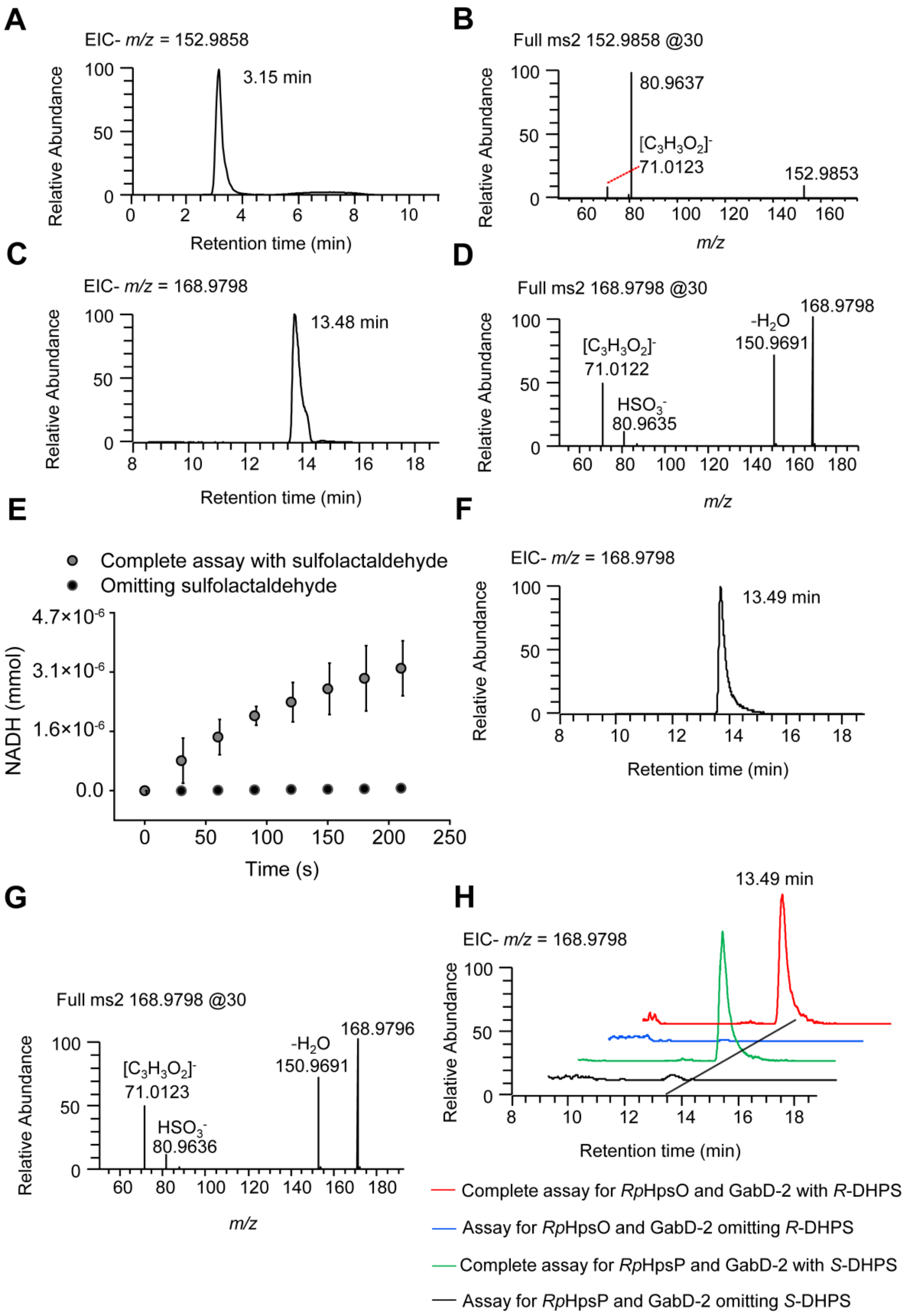
A
EIC- m/z = 152.9858
3.15 min
Relative Abundance
Retention time (min)
B
Full ms2 152.9858 @30
80.9637
[C3H3O2]-
71.0123
152.9853
m/z
C
EIC- m/z = 168.9798
13.48 min
D
Full ms2 168.9798 @30
168.9798
-H2O
150.9691
HSO3-
80.9635
71.0122
E
Complete assay with sulfolactaldehyde
Omitting sulfolactaldehyde
NADH (mmol)
Time (s)
F
EIC- m/z = 168.9798
13.49 min
G
Full ms2 168.9798 @30
168.9796
150.9691
71.0123
80.9636
H
EIC- m/z = 168.9798
13.49 min
Complete assay for RpHpsO and GabD-2 with R-DHPS
Assay for RpHpsO and GabD-2 omitting R-DHPS
Complete assay for RpHpsP and GabD-2 with S-DHPS
Assay for RpHpsP and GabD-2 omitting S-DHPS

Fig. S4 Enzymatic reaction products analysis. (**A**) Extracted ion chromatogram of sulfolactaldehyde standard with *m/z* 152.9858. (**B**) MS/MS fragmentation of sulfolactaldehyde standard with HSO_3^- (*m/z* 81) and $[C_3H_3O_2]^-$ (*m/z* 71) ions. (**C**) Extracted ion chromatogram of sulfolactate standard with *m/z* 168.9798. (**D**) MS/MS fragmentation of sulfolactate standard lead to the loss of a water (-18) and the formation of HSO_3^- (*m/z* 81) and $[C_3H_3O_2]^-$ (*m/z* 71) ions. (**E**) Enzymatic activity assay monitoring NADH formation accompanying sulfolactaldehyde oxidization by GabD-2 (1.0 μM). (**F**) Extracted ion chromatogram of sulfolactaldehyde standard in reaction buffer with addition of GabD-2 to generate sulfolactate. (**G**) MS/MS fragmentation of sulfolactate generated in GabD-2 reaction with sulfolactaldehyde standard lead to the loss of a water (-18) and to the formation of HSO_3^- (*m/z* 81) and $[C_3H_3O_2]^-$ (*m/z* 71) ions. (**H**) Extracted ion chromatogram (*m/z* 168.9798) of enzymatic reaction products. Red line represents the enzymatic reaction containing *Rp*HpsO, GabD-2 and *R*-DHPS, in which GabD-2 oxidized the sulfolactaldehyde, generated from the oxidization of *R*-DHPS by *Rp*HpsO, to sulfolactate. Blue line represents the blank control for *Rp*HpsO and GabD-2 assay, omitting *R*-DHPS. Green line represents the enzymatic reaction containing *Rp*HpsP, GabD-2 and *S*-DHPS, in which GabD-2 oxidized the sulfolactaldehyde, generated from the oxidization of *S*-DHPS by *Rp*HpsP, to sulfolactate. Black line represents the blank control for *Rp*HpsP and GabD-2 assay, omitting *S*-DHPS.

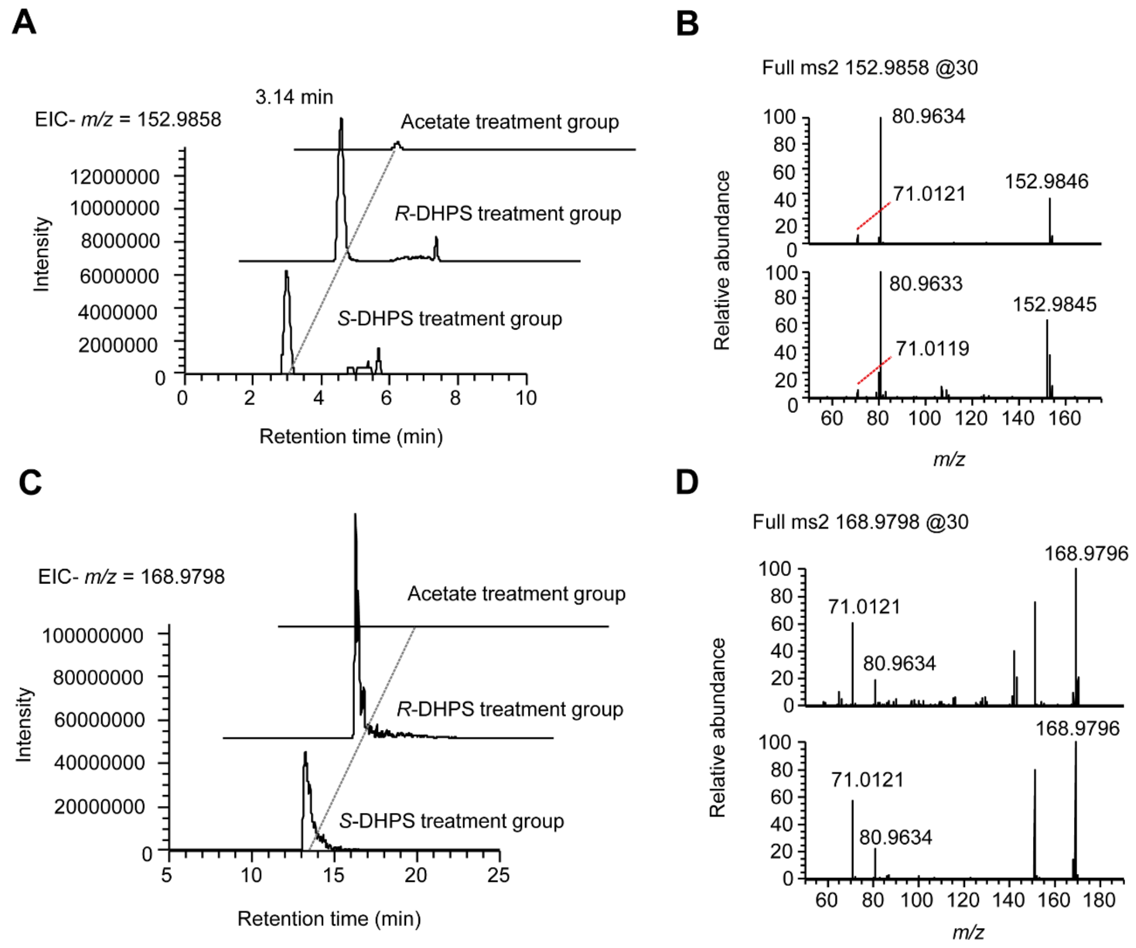

Fig. S5 Intercellular metabolite analysis of *R. pomeroyi* DSS-3. (**A**) Extracted ion chromatogram of sulfolactaldehyde (*m/z* 152.9858) produced from *R. pomeroyi* DSS-3 utilizing *R*- or *S*-DHPS. Cultures of *R. pomeroyi* DSS-3 utilizing acetate were used as a control group. (**B**) MS/MS fragmentations of sulfolactaldehyde generated in *R*-DHPS treatment group (top) and *S*-DHPS treatment group (bottom). (**C**) Extracted ion chromatogram of sulfolactate (*m/z* 168.9798) produced from *R. pomeroyi* DSS-3 utilizing *R*- or *S*-DHPS as carbon source. Cultures of *R. pomeroyi* DSS-3 utilizing acetate were used as a control group. (**D**) MS/MS fragmentations of sulfolactate generated in *R*-DHPS treatment group (top) and *S*-DHPS treatment group (bottom).

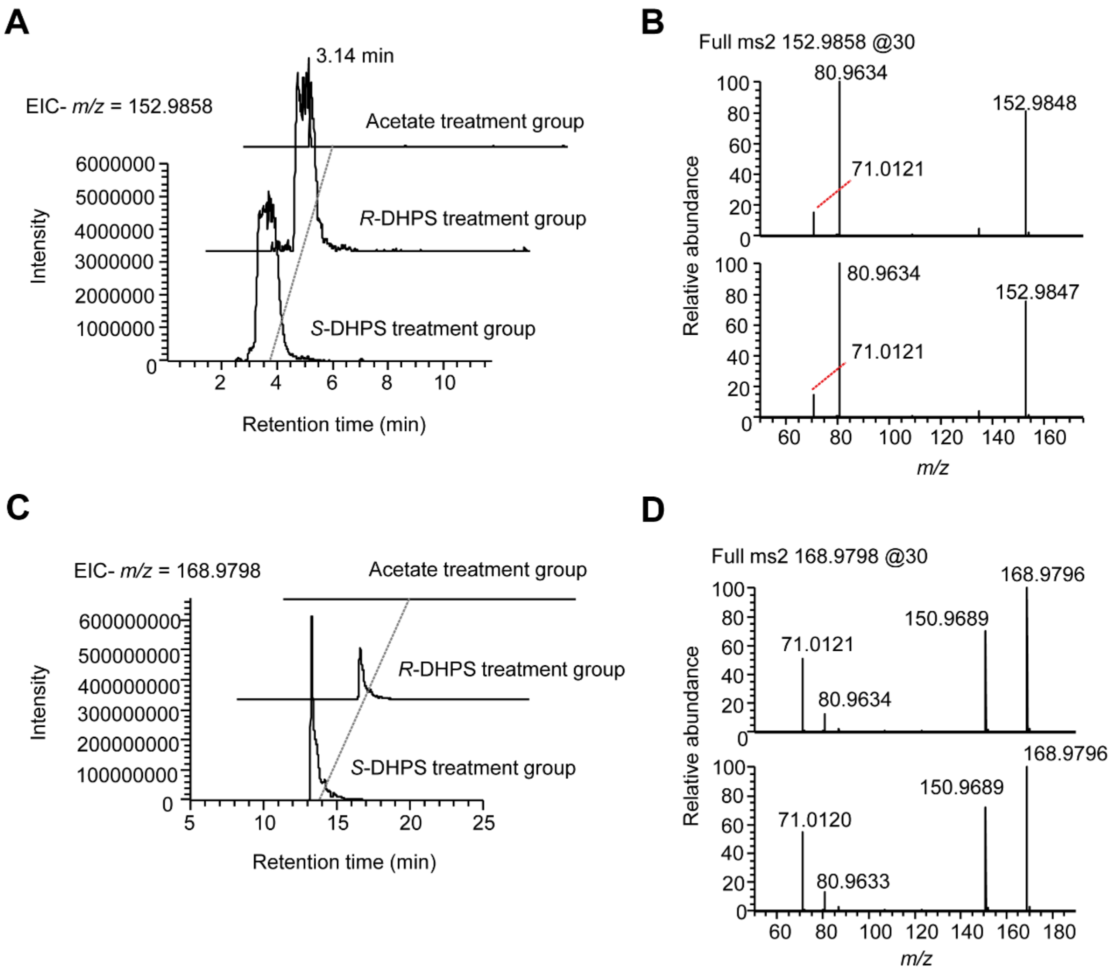

Fig. S6 Intercellular metabolites analysis of *D. shibae* DFL 12. (**A**) Extracted ion chromatogram of sulfolactaldehyde (*m/z* 152.9858) produced from *D. shibae* DFL 12 utilizing *R*- or *S*-DHPS as carbon source. Cultures of *D. shibae* DFL 12 utilizing acetate were used as a control group. (**B**) MS/MS fragmentations of sulfolactaldehyde generated in *R*-DHPS treatment group (top) and *S*-DHPS treatment group (bottom). (**C**) Extracted ion chromatogram of sulfolactate (*m/z* 168.9798) produced from *D. shibae* DFL 12 utilizing *R*- or *S*-DHPS as sole carbon source. Cultures of *D. shibae* DFL 12 utilizing acetate were used as a control group. (**D**) MS/MS fragmentations of sulfolactate generated in *R*-DHPS treatment group (top) and *S*-DHPS treatment group (bottom).

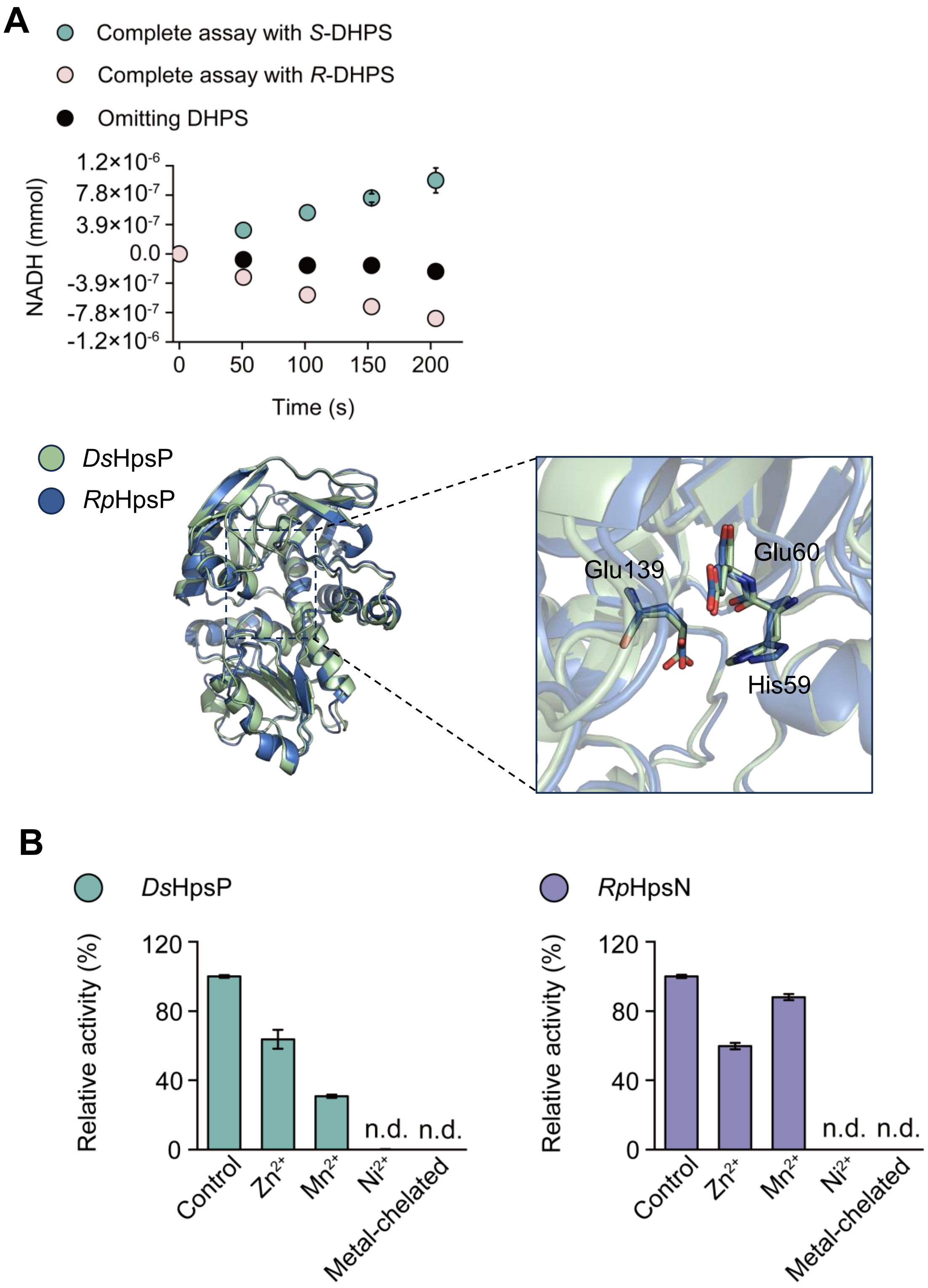
A
Complete assay with S-DHPS
Complete assay with R-DHPS
Omitting DHPS
NADH (mmol)
1.2×10-6
7.8×10-7
3.9×10-7
0.0
-3.9×10-7
-7.8×10-7
-1.2×10-6
0
50
100
150
200
Time (s)
DsHpsP
RpHpsP
Glu139
Glu60
His59
B
DsHpsP
RpHpsN
Relative activity (%)
120
80
40
0
n.d. n.d.
Control
Zn2+
Mn2+
Ni2+
Metal-chelated

Fig. S7 Analysis of structures and enzymatic properties. (**A**) Enzymatic activity assay monitoring NADH formation accompanying *S*-DHPS oxidization by *Ds*HpsP (0.5 μM). The structure alignment of *Ds*HpsP (green) with AlphaFold2-predicted *Rp*HpsP (blue), with a rmsd value of 0.83 Å over 325 residues. The conserved active sites (His59, Glu60 and Glu139) are shown as sticks. (**B**) Effects of metal ions on the enzymatic activity of *Ds*HpsP and *Rp*HpsN.

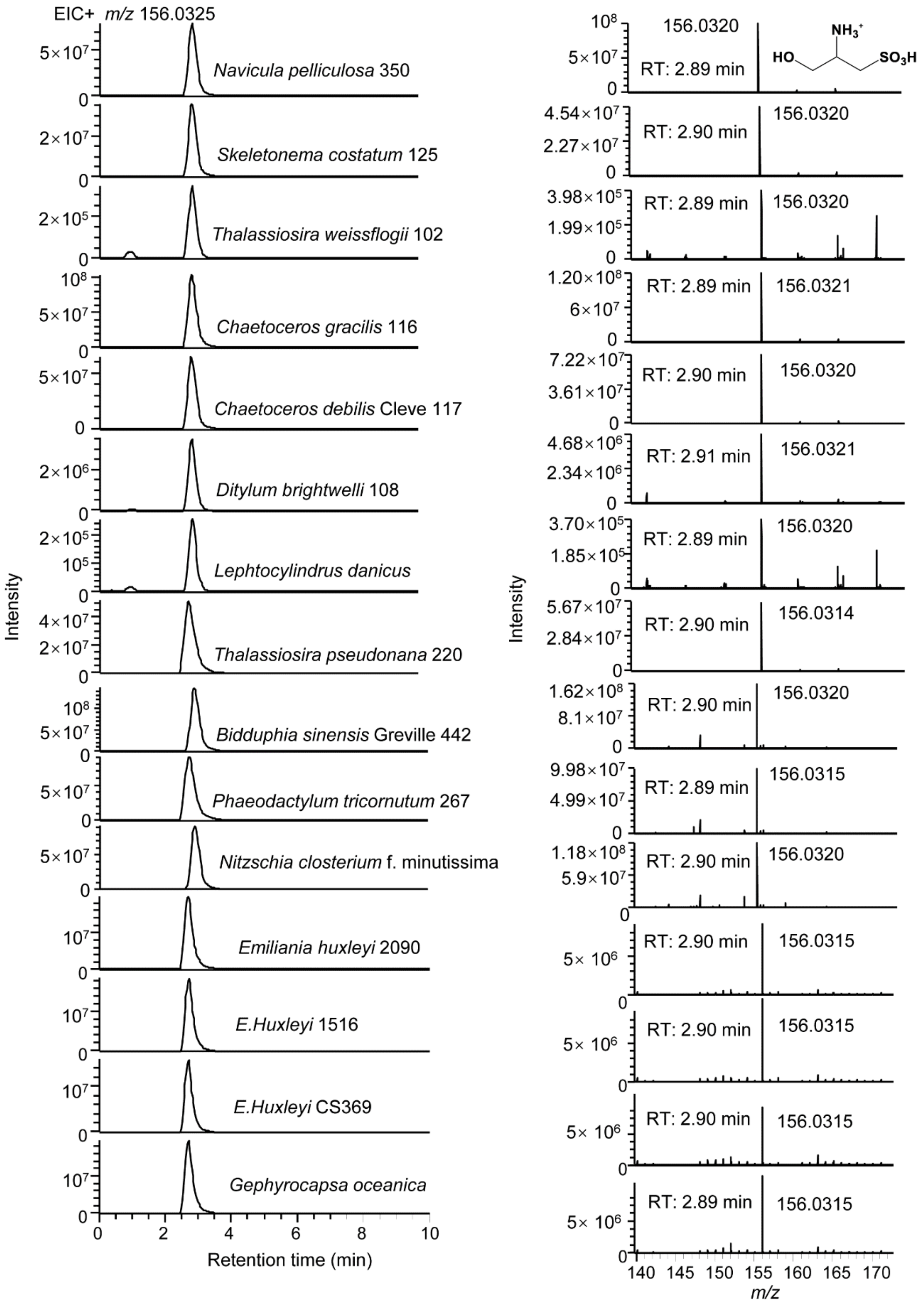

Fig. S8 Extracted ion chromatograms of candidate cysteinolic acid (*m/z* 156.0325) extracted from DHPS-producing diatoms and coccolithophores. Corresponding MS next to the chromatograms in the right panel. RT, retention time.

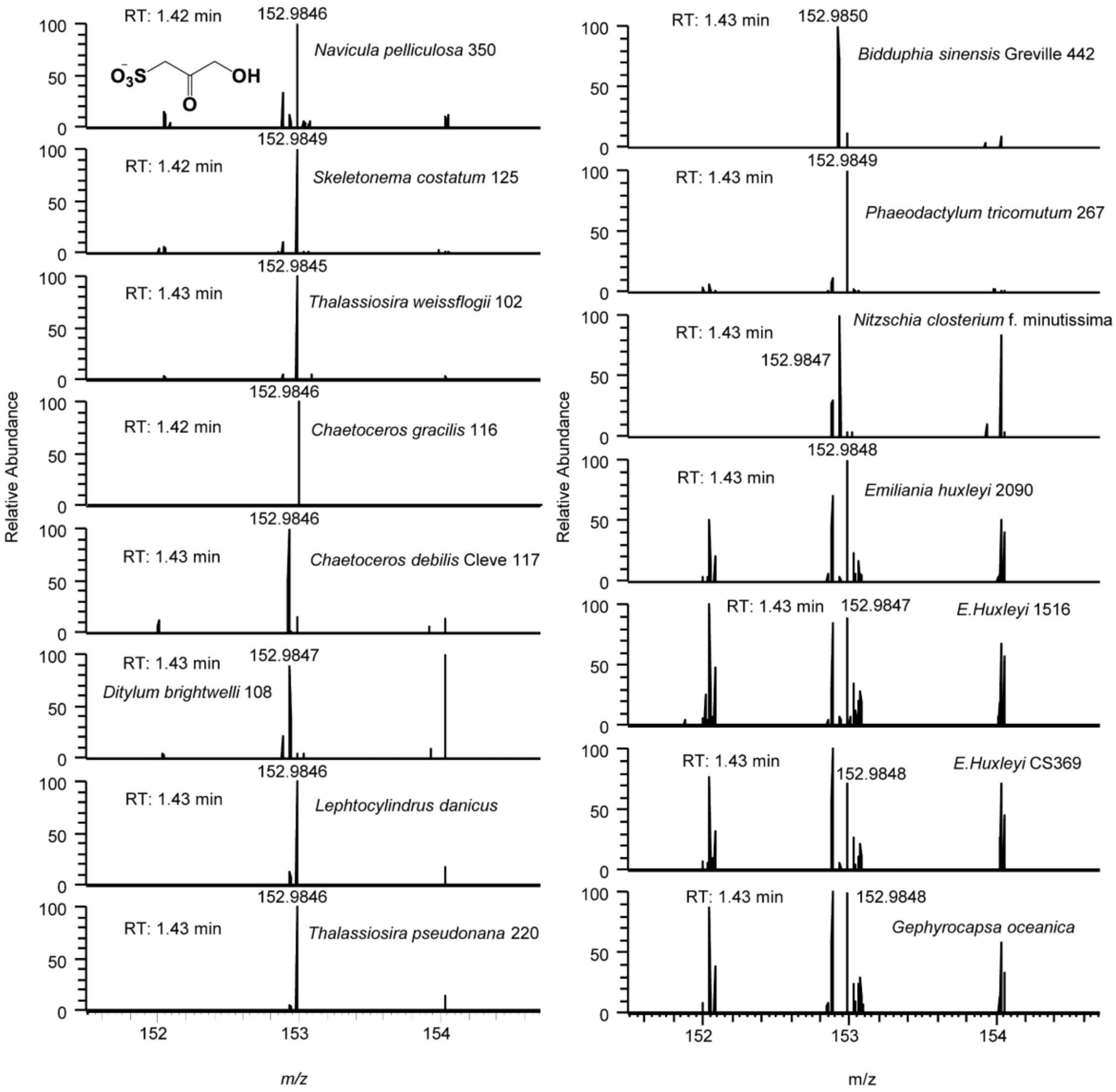

Fig. S9 MS of candidate 2-oxo-3-hydroxy-propane-1-sulfonate (*m/z* 152.9858) extracted from DHPS-producing diatoms and coccolithophores. The chemical structures of cysteionlic acid and 2-oxo-3-hydroxy-propane-1-sulfonate were illustrated. RT, retention time.

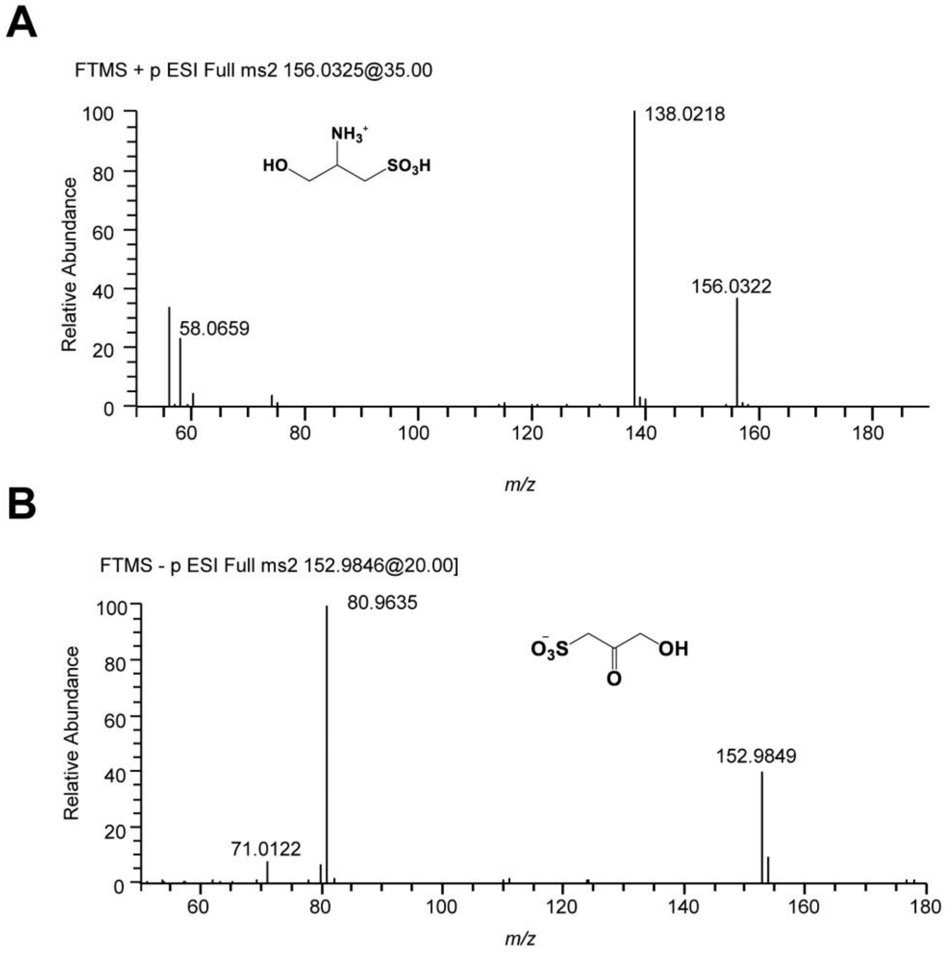

Fig. S10 MS/MS of candidate cysteinolic acid (A) and 2-oxo-3-hydroxy-propane-1-sulfonate (B) from a representative sample of phytoplankton (*N. pelliculosa* 350).

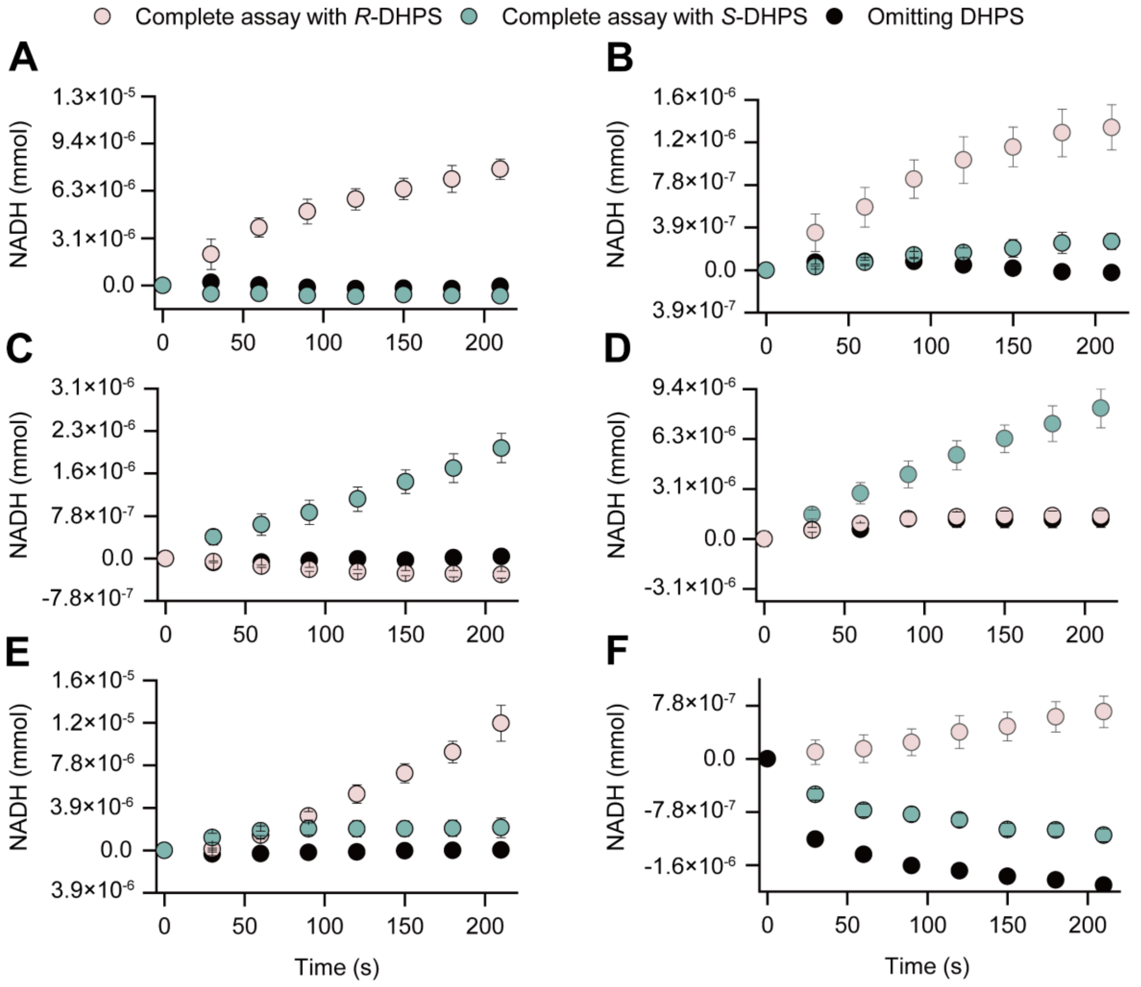

Fig. S11 Enzymatic activity assays monitoring NADH formation accompanying *R*-, *S*-DHPS oxidation by dehydrogenases. (**A**) Enzymatic assay with 0.3 µM *Cp*HpsO from *C. pinatubonensis* JMP134, 10 mM *R*-, *S*-DHPS and 10 mM NAD^+. (**B**) Enzymatic assay with 0.4 µM *Cu*HpsO from *Candidatus* Pelagibacter *ubique* HTCC1002, 10 mM *R*-, *S*-DHPS and 10 mM NAD^+. (**C**) Enzymatic assay with 0.5 µM *Cp*HpsP from *C. pinatubonensis* JMP134, 10 mM *R*-, *S*-DHPS and 10 mM NAD^+. (**D**) Enzymatic assay with 0.5 µM *As*HpsP from *Alphaproteobacteria* sp. SAR11 HIMB5, 10 mM *R*-, *S*-DHPS and 10 mM NAD^+. (**E**) Enzymatic assay with 0.6 µM *Cp*HpsN from *C. pinatubonensis* JMP134, 10 mM *R*-, *S*-DHPS and 10 mM NAD^+. (**F**) Enzymatic assay with 0.2 µM *Cu*HpsN from *C.* Pelagibacter *ubique* HTCC1002, 10 mM *R*-, *S*-DHPS and 10 mM NAD^+. Pink circles indicate the enzymatic assays with *R*-DHPS. Green circles indicate

the enzymatic assays with *S*-DHPS. Black circles indicate blank control omitting the substrates *R*-, *S*-DHPS.

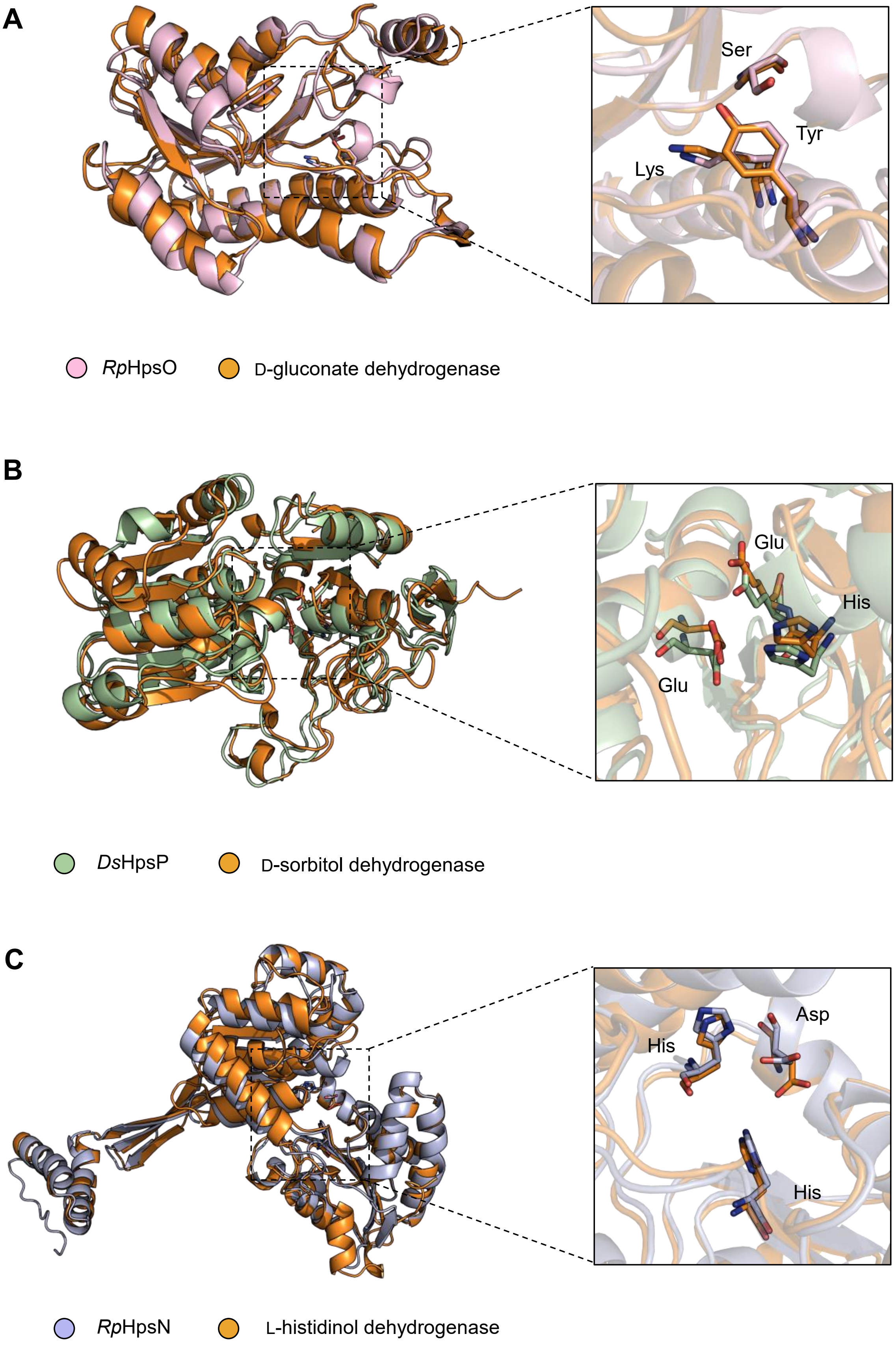
A
Ser
Tyr
Lys
RpHpsO
D-gluconate dehydrogenase
B
Glu
His
Glu
DsHpsP
D-sorbitol dehydrogenase
C
Asp
His
His
RpHpsN
L-histidinol dehydrogenase

Fig. S12 DHPS dehydrogenases structural alignments. (**A**) Structural alignments of *Rp*HpsO with D-gluconate dehydrogenase from *Pectobacterium carotovorum* subsp. carotovorum (PDB ID: 4ZA2). (**B**) Structural alignments of *Ds*HpsP with D-sorbitol dehydrogenase from *Homo sapiens* (PDB ID: 1PL6). (**C**) Structural alignments of *Rp*HpsN with L-histidinol dehydrogenase from *E. coli* MC1061 (PDB ID:1KAE). Conserved active site residues are shown as sticks.

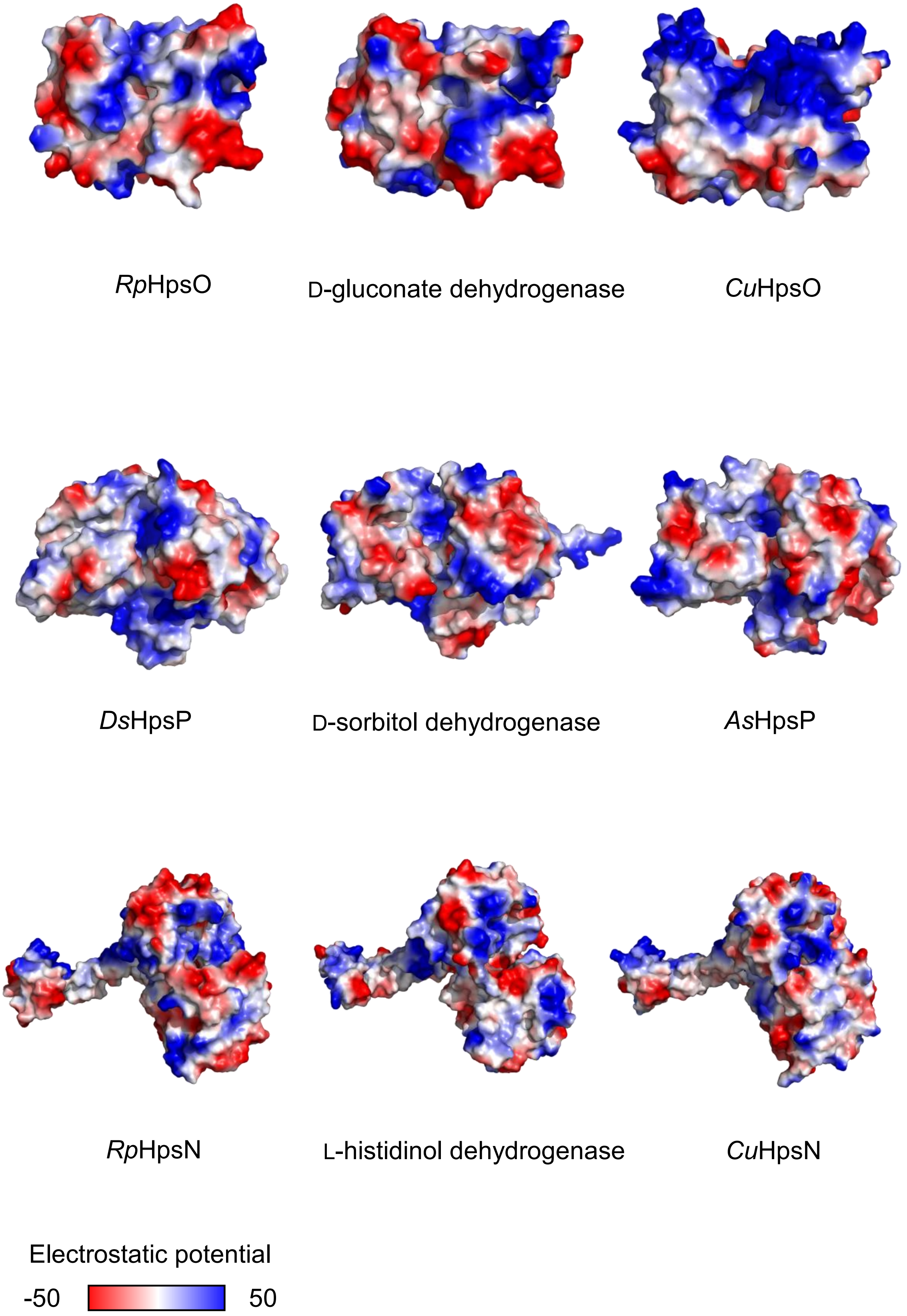

Fig. S13 Electrostatic potentials analysis. Electrostatic potentials of *Rp*HpsO, *Ds*HpsP,

and *Rp*HpsN compared to D-gluconate dehydrogenase from *P. carotovorum* subsp. carotovorum (PDB ID: 4ZA2), D-sorbitol dehydrogenase from *Homo sapiens* (PDB ID: 1PL6), and L-histidinol dehydrogenase from *E. coli* MC1061 (PDB ID:1KAE), as well as homologs from SAR11 bacteria (*Cu*HpsO, *As*HpsP and *Cu*HpsN). SAR11's protein structures were predicted by AlphaFold2.

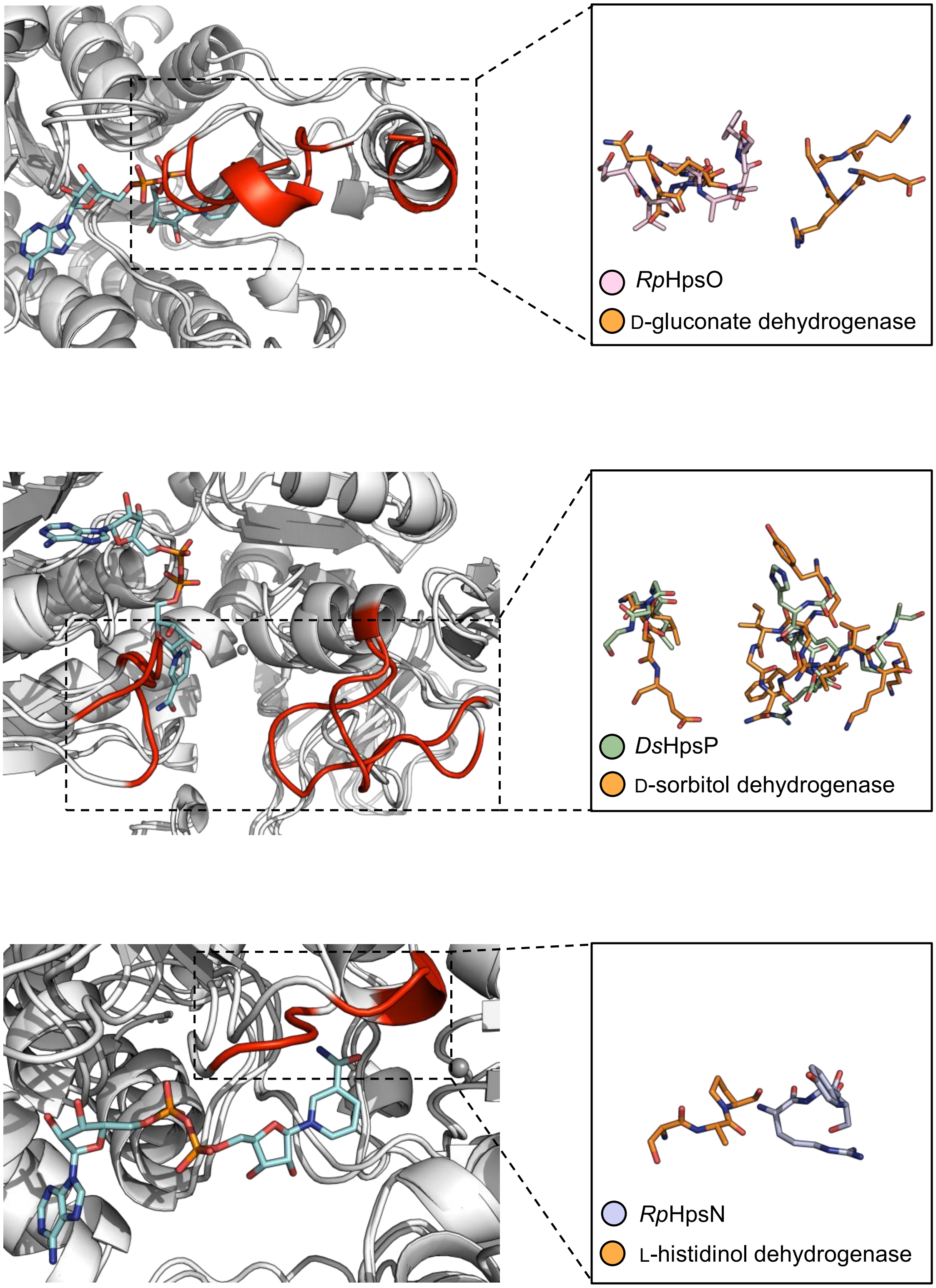

Fig. S14 Structural difference between *Rp*HpsO, *Ds*HpsP, and *Rp*HpsN compared to their homologs. Severely non-conserved regions are colored in red and are shown as sticks.

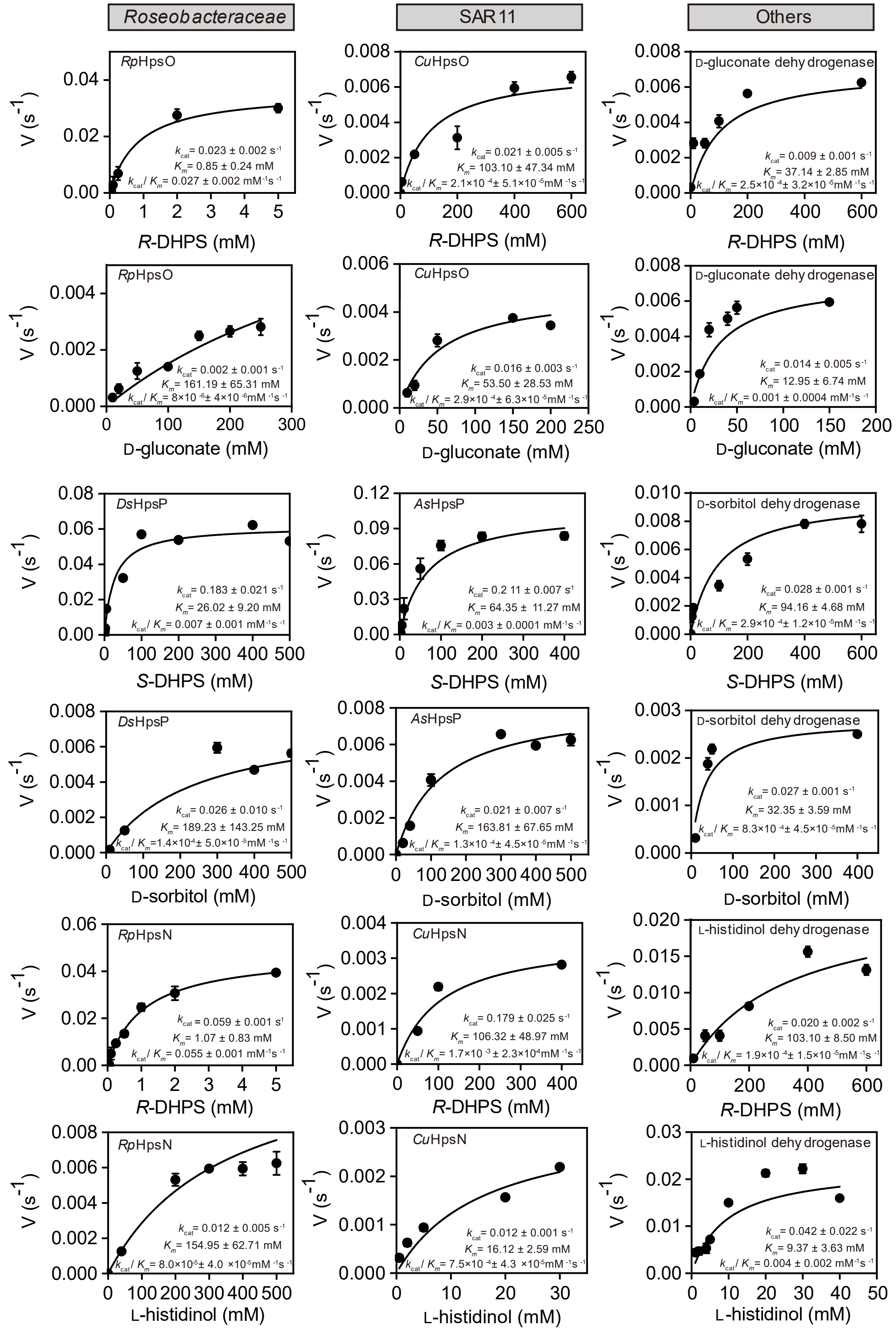
Roseobacteraceae
SAR11
Others
RpHpsO
kcat= 0.023 ± 0.002 s-1
Km= 0.85 ± 0.24 mM
kcat/ Km= 0.027 ± 0.002 mM-1s-1
R-DHPS (mM)
CuHpsO
kcat= 0.021 ± 0.005 s-1
Km= 103.10 ± 47.34 mM
kcat/ Km= 2.1×10-4± 5.1×10-5mM-1s-1
R-DHPS (mM)
D-gluconate dehydrogenase
kcat= 0.009 ± 0.001 s-1
Km= 37.14 ± 2.85 mM
kcat/ Km= 2.5×10-4± 3.2×10-5mM-1s-1
R-DHPS (mM)
RpHpsO
kcat= 0.002 ± 0.001 s-1
Km= 161.19 ± 65.31 mM
kcat/ Km= 8×10-6± 4×10-6mM-1s-1
D-gluconate (mM)
CuHpsO
kcat= 0.016 ± 0.003 s-1
Km= 53.50 ± 28.53 mM
kcat/ Km= 2.9×10-4± 6.3×10-5mM-1s-1
D-gluconate (mM)
D-gluconate dehydrogenase
kcat= 0.014 ± 0.005 s-1
Km= 12.95 ± 6.74 mM
kcat/ Km= 0.001 ± 0.0004 mM-1s-1
D-gluconate (mM)
DsHpsP
kcat= 0.183 ± 0.021 s-1
Km= 26.02 ± 9.20 mM
kcat/ Km= 0.007 ± 0.001 mM-1s-1
S-DHPS (mM)
AsHpsP
kcat= 0.2 11 ± 0.007 s-1
Km= 64.35 ± 11.27 mM
kcat/ Km= 0.003 ± 0.0001 mM-1s-1
S-DHPS (mM)
D-sorbitol dehydrogenase
kcat= 0.028 ± 0.001 s-1
Km= 94.16 ± 4.68 mM
kcat/ Km= 2.9×10-4± 1.2×10-5mM-1s-1
S-DHPS (mM)
DsHpsP
kcat= 0.026 ± 0.010 s-1
Km= 189.23 ± 143.25 mM
kcat/ Km=1.4×10-4± 5.0×10-5mM-1s-1
D-sorbitol (mM)
AsHpsP
kcat= 0.021 ± 0.007 s-1
Km= 163.81 ± 67.65 mM
kcat/ Km= 1.3×10-4± 4.5×10-5mM-1s-1
D-sorbitol (mM)
D-sorbitol dehydrogenase
kcat= 0.027 ± 0.001 s-1
Km= 32.35 ± 3.59 mM
kcat/ Km= 8.3×10-4± 4.5×10-5mM-1s-1
D-sorbitol (mM)
RpHpsN
kcat= 0.059 ± 0.001 s-1
Km= 1.07 ± 0.83 mM
kcat/ Km= 0.055 ± 0.001 mM-1s-1
R-DHPS (mM)
CuHpsN
kcat= 0.179 ± 0.025 s-1
Km= 106.32 ± 48.97 mM
kcat/ Km= 1.7×10-3± 2.3×10-4mM-1s-1
R-DHPS (mM)
L-histidinol dehydrogenase
kcat= 0.020 ± 0.002 s-1
Km= 103.10 ± 8.50 mM
kcat/ Km= 1.9×10-4± 1.5×10-5mM-1s-1
R-DHPS (mM)
RpHpsN
kcat= 0.012 ± 0.005 s-1
Km= 154.95 ± 62.71 mM
kcat/ Km= 8.0×10-5± 4.0 ×10-5mM-1s-1
L-histidinol (mM)
CuHpsN
kcat= 0.012 ± 0.001 s-1
Km= 16.12 ± 2.59 mM
kcat/ Km= 7.5×10-4± 4.3 ×10-5mM-1s-1
L-histidinol (mM)
L-histidinol dehydrogenase
kcat= 0.042 ± 0.022 s-1
Km= 9.37 ± 3.63 mM
kcat/ Km= 0.004 ± 0.002 mM-1s-1
L-histidinol (mM)
V (s-1)

Fig. S15 Michaelis-Menten kinetics of DHPS dehydrogenases. *Roseobacteraceae*, HpsO (1.7 μM), HpsP (0.4 μM) and HpsN (0.6 μM) from *R. pomeroyi* DSS-3 and *D. shibae* DFL 12. SAR11, HpsO (0.2 μM), HpsP (0.5 μM) and HpsN (0.2 μM) from *C.* Pelagibacter *ubique* HTCC1002 and *Alphaproteobacteria* sp. SAR11 HIMB5. Others, D-gluconate dehydrogenase (0.4 μM) from *S. suis* sv. 2 BM411a (Locus tag: Ga0130485_10764), D-sorbitol dehydrogenase (0.12 μM) from *B. subtilis* 168 (Bsubs1_010100003458) and L-histidinol dehydrogenase (0.8 μM) from *E. coli* MC1061 (Ga0125172_11).

Table S1. Primers of site-directed mutagenesis.

Mutants	Primers
*Rp*HpsO (L116A)	5'-GACATCACCCTGACCGCGAACCTGTCGGTG-3'
	5'-GCGGTCAGGGTGATGTCCCAGCCCTCGGGC-3'
*Rp*HpsO (S145A)	5'-ATCGTCAATTTCGCCGCGCTGCAGACAAC-3'
	5'-CGGCGAAATTGACGATCCGGCCCCAGCCG-3'
*Rp*HpsO (Q147A)	5'-CAATTTCGCCTCGCTGGCGACAACCCGCGCC-3'
	5'-GCCAGCGAGGCGAAATTGACGATCCGGCC-3'
*Rp*HpsO (Y158A)	5'-CCCGGTGGCATCGCCGCTGGCGCATCGAAG-3'
	5'-GCGGCGATGCCACCGGGAAAGGCGCGGGTTG-3
*Rp*HpsO (K162A)	5'-GCCTATGGCGCATCGGCGGGCGGCGTGGCGC-3'
	5'-GCCGATGCGCCATAGGCGATGCCACCGGG-3'
*Ds*HpsP (S39A)	5'-GTCGGGATCTGCGGCGCGGACATGCATGC-3'
	5'-CGCCGCAGATCCCGACACTGTCGATGCG-3'
*Ds*HpsP (H59A)	5'-CCGCTGATCCTCGGGGCCGAGGGCGCGGGC-3'
	5'-GCCCCGAGGATCAGCGGGGCAGGGCGGCG-3'
*Ds*HpsP (E60A)	5'-TGATCCTCGGGCACGCGGGCGCGGGCGTG-3'
	5'-GCGTGCCCGAGGATCAGCGGGGCAGGGCG-3'
*Ds*HpsP (E139A)	5'-GAAAGCCGCCCTGGCCGCGCCCGTGGCGGTC-3'
	5'-GCGGCCAGGGCGGCTTTCTCCAGCGGGAC-3'
*Rp*HpsN (P236A)	5'-GACAGGACCGCCGATGCGCATATCGTGAC-3'
	5'-CATCGGCGGTCCTGTCGGCCAGGATCAG-3'
*Rp*HpsN (H320A)	5'-CGCTATGCGCCCGAGGCCCTGACGGTG-3'
	5'-GCCTCGGGCGCATAGCGGTCCGAGGTGG-3'
*Rp*HpsN (D353A)	5'-CCGTCAGCTATGGCGCCAAGGCGGCGGGC-3'
	5'-GCGCCATAGCTGACGGTGCTTTCCTCG-3'
*Rp*HpsN (H360A)	5'-GCGGCGGGCACCAACGCCGTGCTGCCGAC-3'
	5'-GCGTTGGTGCCCGCCGCCTTGTCGCCATAG-3'

Table S2. Data collection and final refinement for crystals of *Rp*HpsO, *Ds*HpsP, and *Rp*HpsN. Statistics for the highest-resolution shell are shown in parentheses.

	*Rp*HpsO
**Data collection**	
Wavelength (Å)	0.9791
Space group	P1
a,b,c (Å)	56.140, 56.156, 76.185
α, β, γ (°)	96.27, 105.96, 90.70
Resolution (Å)	50 – 1.70 (1.73 – 1.70)
No. of observations	93413 (4549)
No. of unique reflections	88739 (6336)
Completeness (%)	95.8 (93.9)
Mean I/δ(I)	17.6 (4.5)
Redundancy	3.4 (3.3)
R_{merge}	0.139 (0.356)
R_{work} /R_{free}	0.1693/0.1866
**No. atoms**	
Water	1081
**B-factors**	
Protein	18.45
Water	29.28
**Ramachandran**	
Favored (%)	97.68
Allowed (%)	2.32
Outlier (%)	0
**RMSD deviations**	
Bond lengths (Å)	0.006
Bond angles (°)	0.95

	*Ds*HpsP
**Data collection**	
Wavelength (Å)	0.9791
Space group	P21
a,b,c (Å)	42.183, 290.538, 54.277
α, β, γ (°)	90.00, 98.38, 90.00
Resolution (Å)	46.96 - 2.30 (2.38 - 2.30)
No. of observations	390244 (40150)
No. of unique reflections	56544 (5608)
Completeness (%)	99.18 (98.54)
Mean I/δ(I)	8.79 (4.42)
Redundancy	6.9 (7.2)
R_{merge}	0.1744 (0.3612)
$R_{p.i.m}$	0.07174 (0.1443)
R_{work} /R_{free}	0.2076/0.2563
**No. atoms**	
Protein	10127
Water	599
**B-factors**	
Protein	37.51
Water	21.85
**Ramachandran**	
Favored (%)	95.5
Allowed (%)	3.6
Outlier (%)	0.6
**RMSD deviations**	
Bond lengths (Å)	0.014
Bond angles (°)	1.82

	*Rp*HpsN
**Data collection**	
Wavelength (Å)	0.9791
Space group	P 2 21 21
a,b,c (Å)	68.045, 122.865, 210.235
α, β, γ (°)	90.00 90.00 90.00
Resolution (Å)	46.2- 2.90 (3.00 - 2.90)
No. of observations	514849 (54213)
No. of unique reflections	39957 (3932)
Completeness (%)	99.40 (99.90)
Mean I/δ(I)	20.60 (3.31)
Redundancy	12.9 (13.8)
R_{merge}	0.2008 (1.191)
$R_{p.i.m}$	0.0594 (0.3293)
R_{work} /R_{free}	0.242/ 0.279
**No. atoms**	
Protein	11125
Ligand/ion	25
**B-factors**	
Protein	56.47
Ligand/ion	89.6
**Ramachandran**	
Favored (%)	94
Allowed (%)	5.2
Outlier (%)	0.8
**RMSD deviations**	
Bond lengths (Å)	0.015
Bond angles (°)	1.85

Table S3. Michaelis-Menten kinetics of *Rp*HpsN, *Rp*HpsO, and *Ds*HpsP using $NADP^+$ as a cofactor.

	k_{cat} (s^{-1})	K_m (mM)	k_{cat}/K_m (mM^{-1} s^{1})
*Rp*HpsN	0.0088 ± 0.0015	8.4 ± 2.5	0.0011 ± 0.0002
*Rp*HpsO	0.0020 ± 0.0002	2.0 ± 0.5	0.0010 ± 0.0002
*Ds*HpsP	0.014 ± 0.004	3.2 ± 1.7	0.0047 ± 0.0012

Table S4. Metal ions in *Rp*HpsN and *Ds*HpsP.

	Protein concentration (μM)	Zn^{2+} (μM)	Mn^{2+} (μM)	Ni^{2+} (μM)
*Rp*HpsN	11.4	138.4	12.2	3.1
*Ds*HpsP	5.0	52.4	3.1	2.1

Table S5. The correlation analysis among gene expression from *Roseobacteraceae* and SAR11 in *Tara* Oceans with environmental factors and algae.

		Spearman's *r*						
		Diatom	Haptophytes	Dianoflagellate	*Synechococcus*	*Prochlorococcus*	Chlorophyll *a*	Temperature
Roseobacteraceae	*hpsO*	0.56	0.17	-0.20	-0.43	-0.07	0.45	-0.32
	hpsP	0.53	0.21	-0.12	-0.44	-0.05	0.56	-0.40
	hpsN	0.58	0.22	-0.13	-0.52	-0.18	0.62	-0.53
		p value						
		Diatom	Haptophytes	Dianoflagellate	*Synechococcus*	*Prochlorococcus*	Chlorophyll *a*	Temperature
Roseobacteraceae	*hpsO*	7.7E-12	1.9E-2	1.7E-1	1.3E-08	8.4E-2	1.1E-14	5.0E-13
	hpsP	4.3E-11	7.2E-2	3.1E-2	4.2E-06	5.3E-1	1.1E-07	2.7E-05
	hpsN	6.0-10	2.5E-2	2.0E-1	1.9E-06	6.6E-1	5.7E-12	9.9E-08

		Spearman's *r*						
		Diatom	Haptophytes	Dianoflagellate	*Synechococcus*	*Prochlorococcus*	Chlorophyll *a*	Temperature
SAR11	*hpsO*	-0.73	0.08	0.08	0.66	0.40	-0.39	0.49
	hpsP	-0.60	0.16	0.05	0.66	0.40	-0.25	0.52
	hpsN	-0.30	0.40	0.07	0.36	0.23	0.09	0.26
		p value						
		Diatom	Haptophytes	Dianoflagellate	*Synechococcus*	*Prochlorococcus*	Chlorophyll *a*	Temperature
SAR11	*hpsO*	8.0E-21	3.6E-1	3.9E-1	7.2E-15	8.6E-05	6.3E-06	2.8E-11
	hpsP	5.7E-13	8.6E-2	5.9E-1	8.2E-15	7.2E-05	4.3E-3	1.1E-12
	hpsN	9.0E-4	9.3E-06	4.8E-1	2.0E-4	2.8E-2	3.3E-1	8.0E-4
